# Supplementary material for: The apparent exponential radiation of Phanerozoic land vertebrates is an artefact of spatial sampling biases
Source: Proc Biol Sci. 2020 Apr 8;287(1924):20200372. doi: 10.1098/rspb.2020.0372 (PMC7209054; doi:10.1098/rspb.2020.0372)
Supplement: Supplementary methods and results [file rspb20200372supp1.docx]

# Supplementary Information for ‘The apparent exponential radiation of Phanerozoic land vertebrates is an artefact of spatial sampling biases’

Roger A. Close^1,*^, Roger B. J. Benson^2^, John Alroy^3^, Matthew T. Carrano^4^, Terri J. Cleary^1^, Emma M. Dunne^1^, Philip D. Mannion^5^, Mark D. Uhen^6^, Richard J. Butler^1,*^

# Supplementary Methods

# *Note on spatial subsampling procedure*. We use the spatial distribution of fossil localities with well-defined palaeocoordinates to quantify the palaeogeographic extent of the known fossil record for each interval. The strength of the correlation between geographic spread and estimated richness is very great (Figs 1 and S6), and is unlikely to be the result of errors. Minor errors would primarily arise from recording modern-day geographic coordinates inaccurately in the Paleobiology Database, and from tectonic rotations used to recover paleocoordinates. However, for most of the standardised palaeogeographic regions that we analyse (i.e., subsamples of fossil localities with approximately equal geographic extents), the localities come from regions of the globe that are linked on a single tectonic plate that moves as a rigid unit. Therefore, the error associated with these estimates are, for our purposes, negligible.

# Supplementary Results

*Model-fitting with additional richness estimators*. Model-selection and fitting results for other richness estimators are given in Tables S5–S6 (SQS with GCR), S7–S8 (face-value species counts), S9–S10 (squares) and S11–S12 (Chao 2). Results are highly congruent for all richness estimators, with the “Time * Pre/Post-K/Pg phase” model receiving highest support. In all models that include phase and time as an interaction term, this is due to a significant decrease in richness through the Cenozoic (Tables S8, S10 and S12; Fig. S9).

**Supplementary References**

47. Hyndman, R. J. & Khandakar, Y. 2008 Automatic time series forecasting: the forecast package for R. *J. Stat. Soft.* **27**. (doi:10.18637/jss.v027.i03)

**
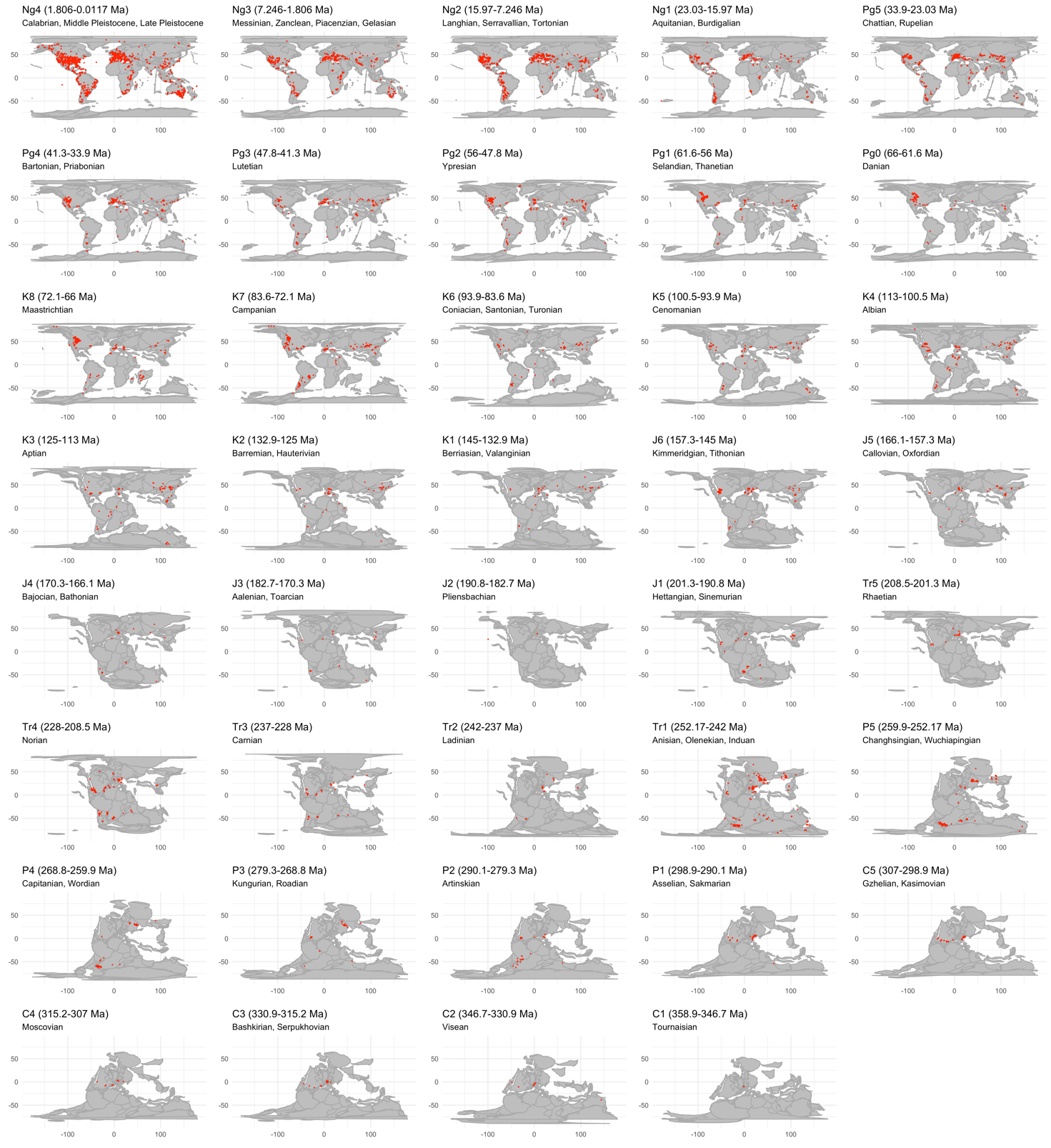
**

**Fig. S1.** Distribution of non-flying tetrapod fossil localities through the Phanerozoic, using equal-length time bins.


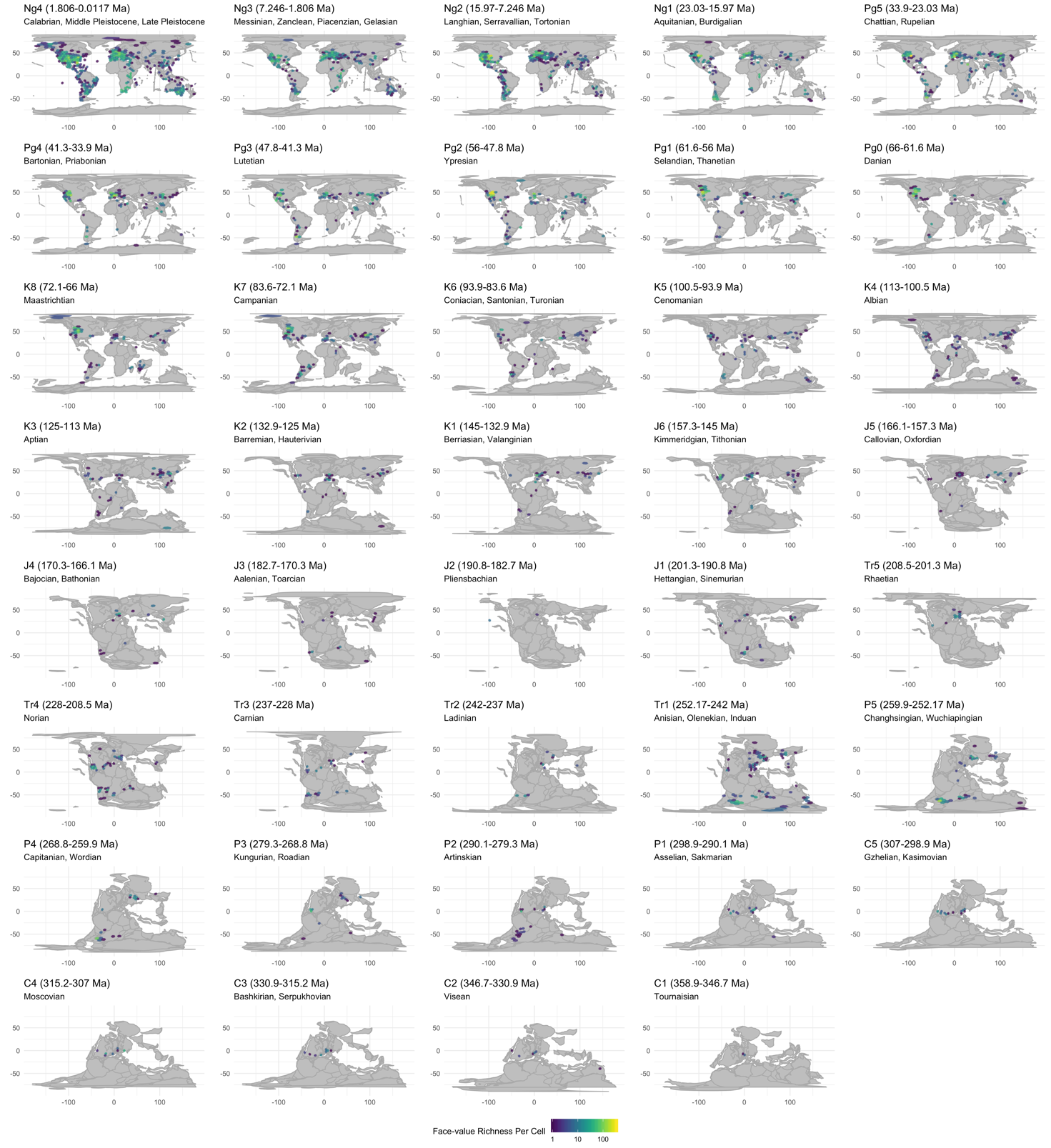


**Fig. S2.** Distribution of equal-sized hexagonal/pentagonal grid cells with 500 km spacings (between cell midpoints) containing occurrences of non-flying tetrapod fossils through the Phanerozoic, using equal-length time bins. Colours represent face-value species counts per cell.

**
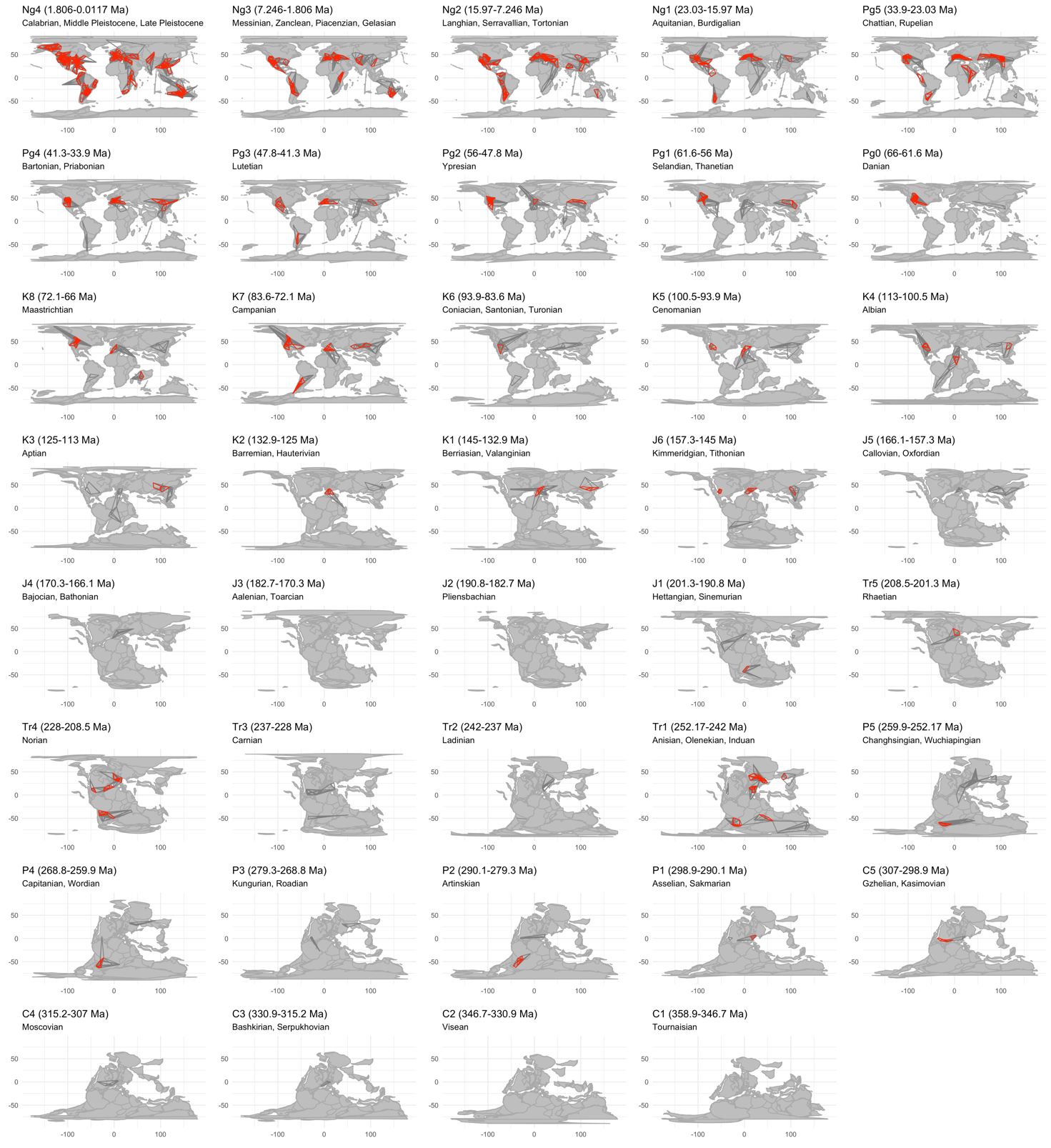
**

**Fig. S3.** Distribution of subsampled spatial regions sampling non-flying tetrapod fossils through the Phanerozoic, using equal-length time bins. Spatial regions meeting spatial standardisation criteria for 2000 km MST lengths (see Methods for full list of criteria) are in red, and those not meeting these criteria are in grey.


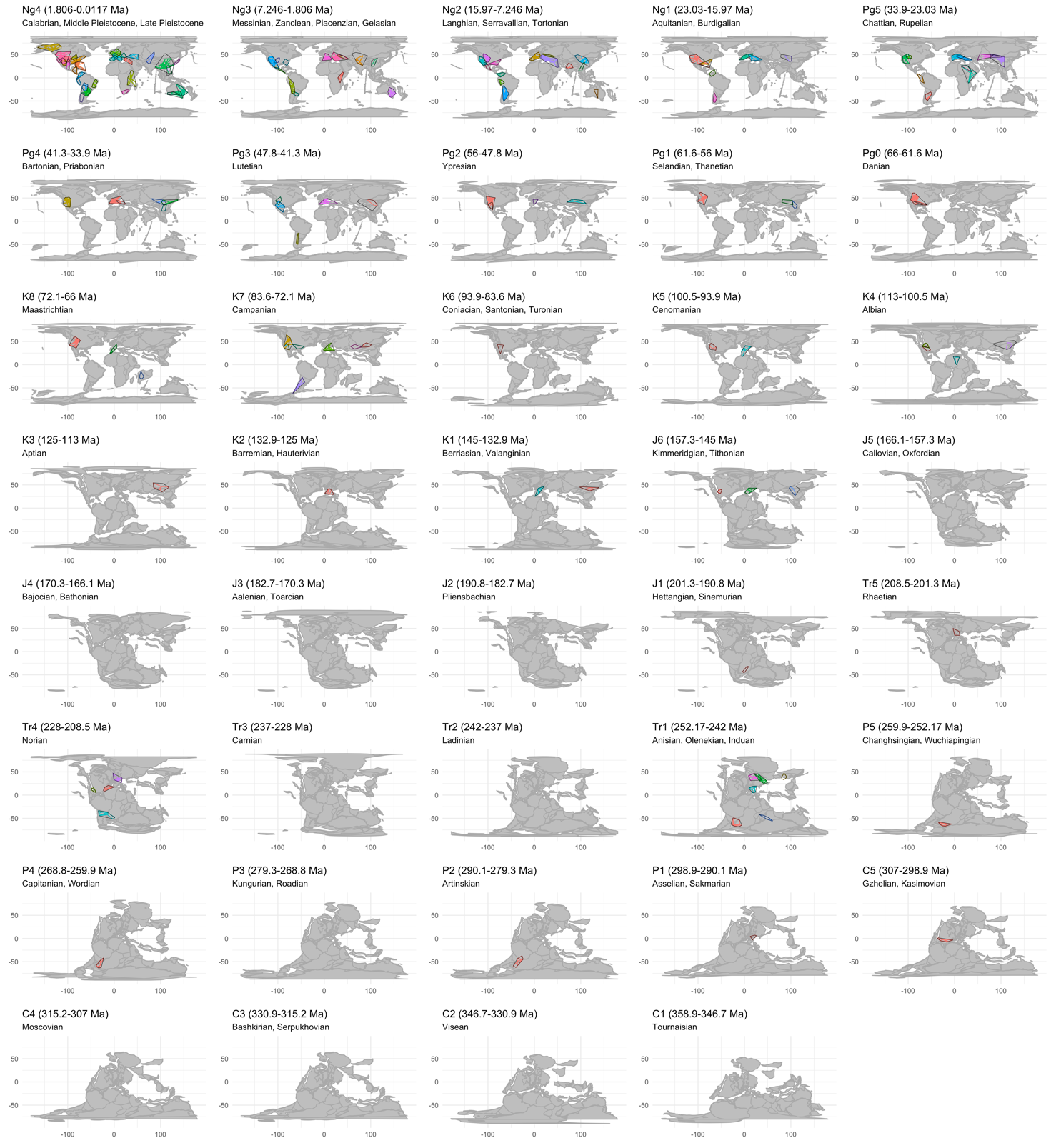


**Fig. S4.** Clusters of subsampled spatial regions (2000 km MST length) for non-flying tetrapods through the Phanerozoic, using equal-length time bins. Colours differentiate clusters.


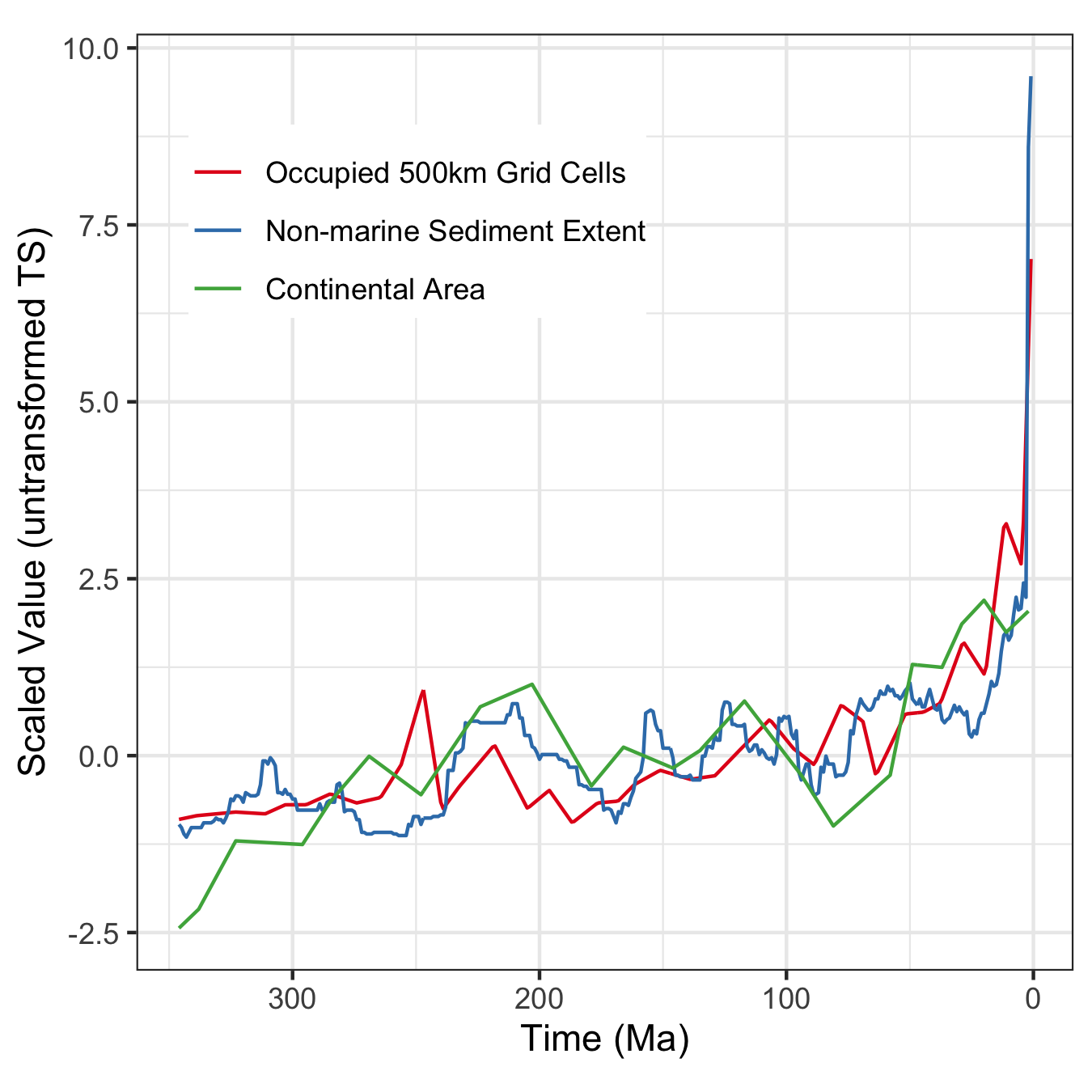


**Fig. S5.** Time series (scaled to unit variance and centred) for the palaeogeographic spread of the worldwide non-flying terrestrial tetrapod fossil record (occupied equal-area grid cells with 500 km spacings), and estimates of continental area (from Cao et al. [36]) and non-marine sediment extent (derived from Macrostrat by [40]). Only non-marine sediment extent mirrors palaeogeographic spread in rising sharply during the Neogene–Recent, and increases in continental area over the same interval are much smaller.


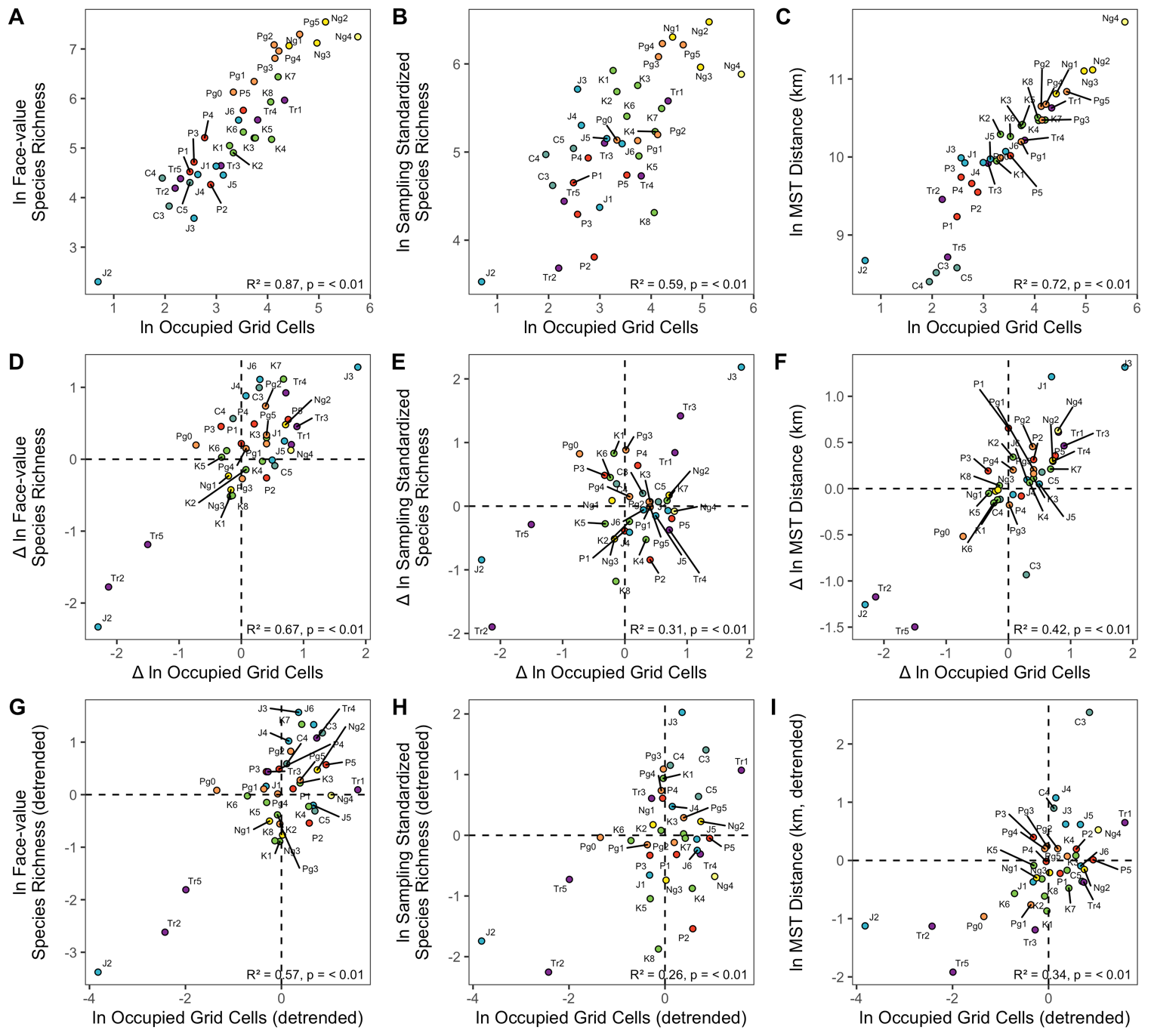


**Fig. S6.** Bivariate relationships between the palaeogeographic spread of the worldwide non-marine, non-flying tetrapod fossil record, quantified using per-bin counts of occupied equal-area grid cells with 500 km spacings ([36]) and other key variables. (A–B) Raw (i.e. not detrended or differenced) relationships between time series occupied grid cell counts and “global” tetrapod species richness estimates (face-value counts of species, and sampling standardised SQS richness at quorum = 0.6). (D–E) Corresponding first-differenced relationships. (G–H) Corresponding relationships for time series detrended with ARIMA models (using the R function auto.arima() in the package forecast [47]). (C, F, I) Relationships between palaeogeographic spread quantified using occupied grid cells, and using MST length. All variables log-transformed. Datapoints for C1 and C2 removed as outliers.


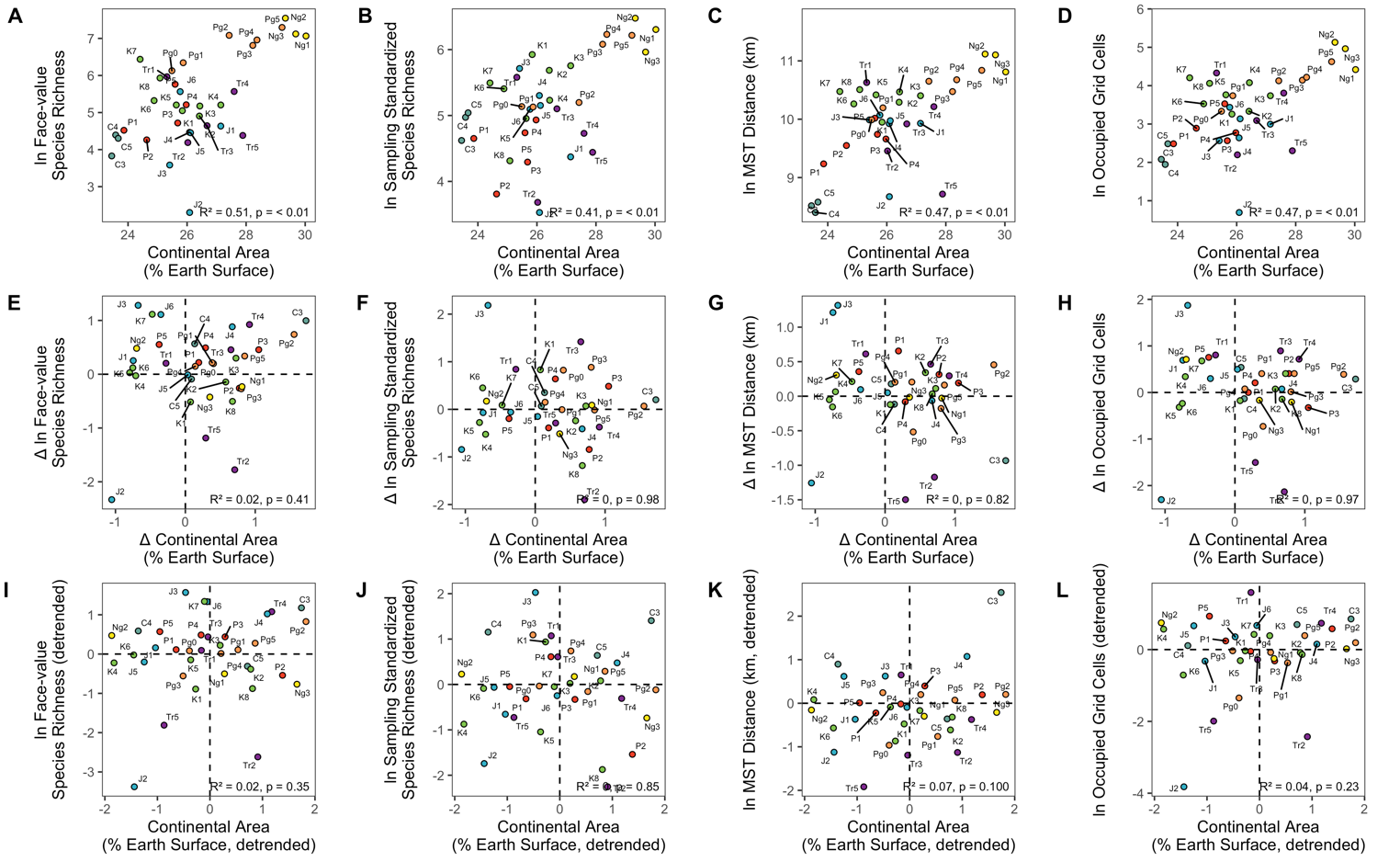


**Fig. S7.** Bivariate relationships between an estimate of continental area through the Phanerozoic ([36]) and other key variables. (A–D) Raw (i.e. not detrended or differenced) relationships between time series of continental area, “global” tetrapod species richness estimates, and the palaeogeographic spread of their fossil record. (E–H) Corresponding first-differenced relationships. (I–L) Corresponding relationships for time series detrended with ARIMA models (using the R function auto.arima() in the package forecast [47]). Datapoints for C1 and C2 removed as outliers. Although relationships using ‘raw’ time series are significant, accounting for spurious time series effects renders them non-significant.


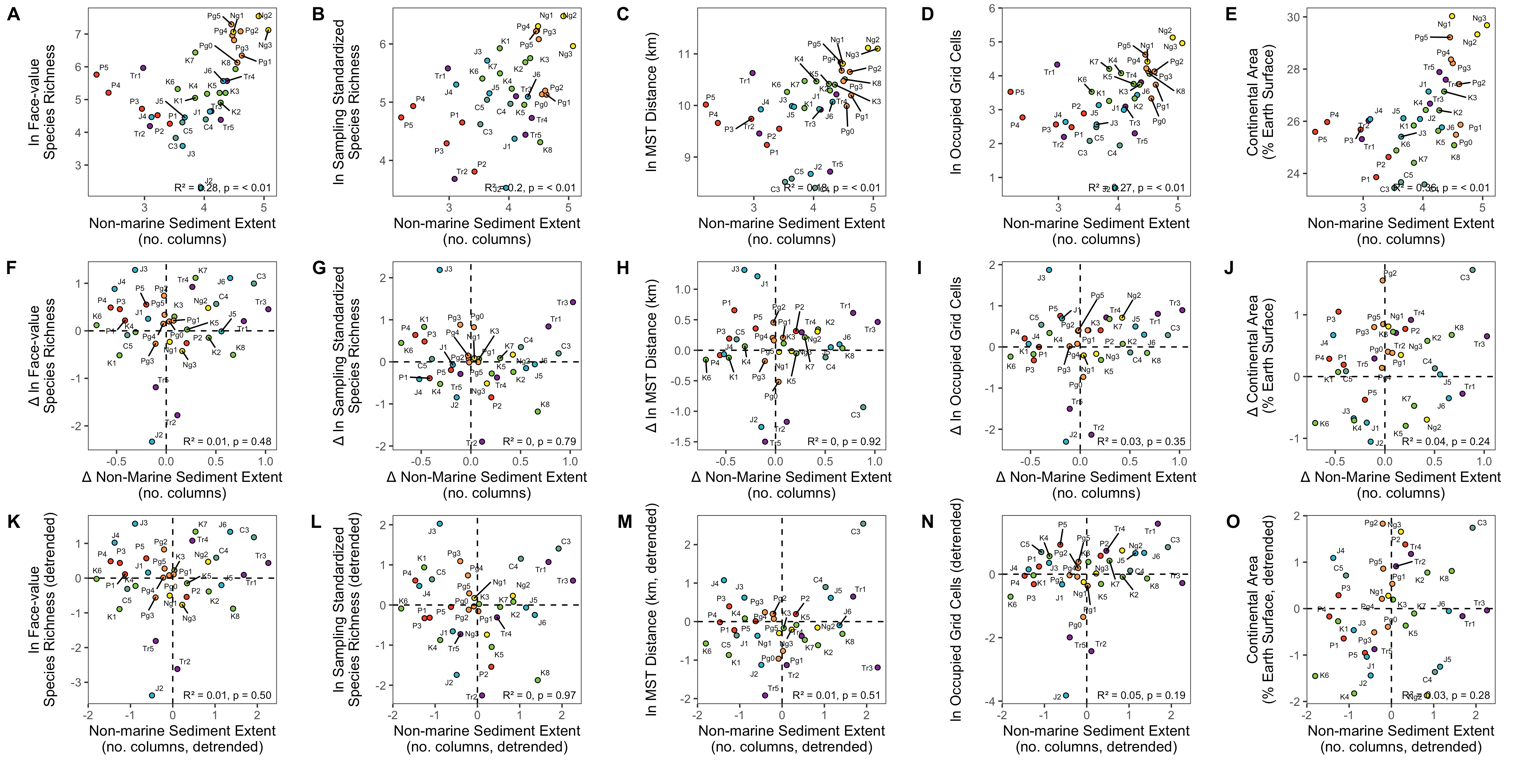


**Fig. S8.** Bivariate relationships between non-marine sediment extent (derived from the Macrostrat database (http://www.macrostrat.org), via Peters and Husson [40]) and other key variables. (A–E) Raw (i.e. not detrended or differenced) relationships between time series of non-marine sediment extent and diversity, palaeogeographic spread and continental area. (F–J) Corresponding first-differenced relationships. (K–O) Corresponding relationships for time series detrended with ARIMA models (using the R function auto.arima() in the package forecast [47]). Datapoints for C1 and C2 removed as outliers. Although relationships using ‘raw’ time series are significant, accounting for spurious time series effects renders them non-significant.

**Fig. S9.** Linear models of ln richness as a function of time within pre- and post-K/Pg diversification phases, for face-value species counts (= raw or uncorrected richness; i.e., not sampling-standardised), squares’ extrapolated species richness and SQS richness (quorum = 0.6). No grid-cell rarefaction used (GCR = off). Shaded envelopes denote 95% confidence intervals for regression slopes. Regressions for the pre-K/Pg phase are never significant, but those for the post-K/Pg phase are sometimes significant, with a positive slope (indicating a statistically significant decline in diversity towards the present).


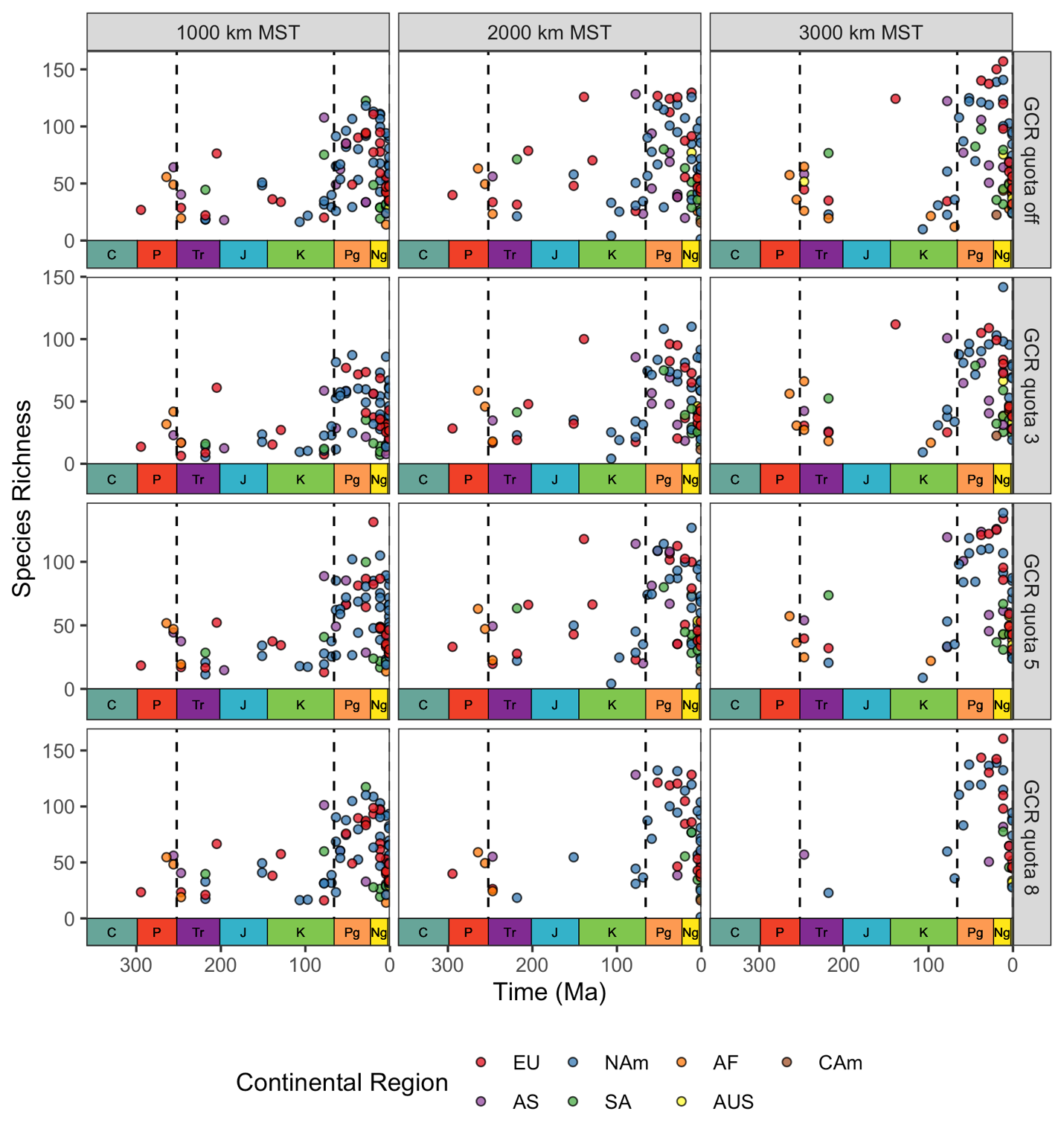


**Fig. S10.** Effects of using a grid-cell rarefaction procedure (using quotas of 3, 5 and 8 occupied cells per 1000 km of summed MST distance) prior to computing SQS richness estimates (quorum = 0.6) on spatially-standardised regions. GCR algorithm not used for “GCR quota = off”. As the GCR quota is raised, increasingly fewer suitable regions are available from pre-Cenozoic intervals.

**Supplementary Tables**

# Table S1.

Definitions of composite time bins of approximately equal length.

| bin | stages | LAD | FAD | midpoint | duration |
| --- | --- | --- | --- | --- | --- |
| Ng4 | Calabrian, Middle Pleistocene, Late Pleistocene | 0.0117 | 1.806 | 0.90885 | 1.7943 |
| Ng3 | Messinian, Zanclean, Piacenzian, Gelasian | 1.8060 | 7.246 | 4.52600 | 5.4400 |
| Ng2 | Langhian, Serravallian, Tortonian | 7.2460 | 15.970 | 11.60800 | 8.7240 |
| Ng1 | Aquitanian, Burdigalian | 15.9700 | 23.030 | 19.50000 | 7.0600 |
| Pg5 | Chattian, Rupelian | 23.0300 | 33.900 | 28.46500 | 10.8700 |
| Pg4 | Bartonian, Priabonian | 33.9000 | 41.300 | 37.60000 | 7.4000 |
| Pg3 | Lutetian | 41.3000 | 47.800 | 44.55000 | 6.5000 |
| Pg2 | Ypresian | 47.8000 | 56.000 | 51.90000 | 8.2000 |
| Pg1 | Selandian, Thanetian | 56.0000 | 61.600 | 58.80000 | 5.6000 |
| Pg0 | Danian | 61.6000 | 66.000 | 63.80000 | 4.4000 |
| K8 | Maastrichtian | 66.0000 | 72.100 | 69.05000 | 6.1000 |
| K7 | Campanian | 72.1000 | 83.600 | 77.85000 | 11.5000 |
| K6 | Coniacian, Santonian, Turonian | 83.6000 | 93.900 | 88.75000 | 10.3000 |
| K5 | Cenomanian | 93.9000 | 100.500 | 97.20000 | 6.6000 |
| K4 | Albian | 100.5000 | 113.000 | 106.75000 | 12.5000 |
| K3 | Aptian | 113.0000 | 125.000 | 119.00000 | 12.0000 |
| K2 | Barremian, Hauterivian | 125.0000 | 132.900 | 128.95000 | 7.9000 |
| K1 | Berriasian, Valanginian | 132.9000 | 145.000 | 138.95000 | 12.1000 |
| J6 | Kimmeridgian, Tithonian | 145.0000 | 157.300 | 151.15000 | 12.3000 |
| J5 | Callovian, Oxfordian | 157.3000 | 166.100 | 161.70000 | 8.8000 |
| J4 | Bajocian, Bathonian | 166.1000 | 170.300 | 168.20000 | 4.2000 |
| J3 | Aalenian, Toarcian | 170.3000 | 182.700 | 176.50000 | 12.4000 |
| J2 | Pliensbachian | 182.7000 | 190.800 | 186.75000 | 8.1000 |
| J1 | Hettangian, Sinemurian | 190.8000 | 201.300 | 196.05000 | 10.5000 |
| Tr5 | Rhaetian | 201.3000 | 208.500 | 204.90000 | 7.2000 |
| Tr4 | Norian | 208.5000 | 228.000 | 218.25000 | 19.5000 |
| Tr3 | Carnian | 228.0000 | 237.000 | 232.50000 | 9.0000 |
| Tr2 | Ladinian | 237.0000 | 242.000 | 239.50000 | 5.0000 |
| Tr1 | Anisian, Olenekian, Induan | 242.0000 | 252.170 | 247.08500 | 10.1700 |
| P5 | Changhsingian, Wuchiapingian | 252.1700 | 259.900 | 256.03500 | 7.7300 |
| P4 | Capitanian, Wordian | 259.9000 | 268.800 | 264.35000 | 8.9000 |
| P3 | Kungurian, Roadian | 268.8000 | 279.300 | 274.05000 | 10.5000 |
| P2 | Artinskian | 279.3000 | 290.100 | 284.70000 | 10.8000 |
| P1 | Asselian, Sakmarian | 290.1000 | 298.900 | 294.50000 | 8.8000 |
| C5 | Gzhelian, Kasimovian | 298.9000 | 307.000 | 302.95000 | 8.1000 |
| C4 | Moscovian | 307.0000 | 315.200 | 311.10000 | 8.2000 |
| C3 | Bashkirian, Serpukhovian | 315.2000 | 330.900 | 323.05000 | 15.7000 |
| C2 | Visean | 330.9000 | 346.700 | 338.80000 | 15.8000 |
| C1 | Tournaisian | 346.7000 | 358.900 | 352.80000 | 12.2000 |

**Table S2.** Model selection using the second-order Akaike information criterion (AICc) to compare fits of linear models of spatially-standardised non-flying terrestrial species richness (SQS, quorum = 0.6; 1000–4000 km MST distance, GCR = off) as a function of time and diversification phase.

| **model** | **df** | **logLik** | **AICc** | **delta AICc** | **weights** | **cumulative weights** | **evidence ratio** |
| --- | --- | --- | --- | --- | --- | --- | --- |
| **1000 km summed MST distance** | | | | | | | |
| Time * Phase | 4 | -71.6 | 154 | 0.00 | 5.86e-01 | 0.586 | 1.00 |
| Phase Only | 2 | -74.5 | 155 | 1.47 | 2.81e-01 | 0.867 | 2.09 |
| Time + Phase | 3 | -74.2 | 157 | 3.08 | 1.25e-01 | 0.992 | 4.69 |
| Time Only | 2 | -78.1 | 162 | 8.68 | 7.64e-03 | 1.000 | 76.70 |
| Intercept Only | 1 | -82.5 | 169 | 15.40 | 2.66e-04 | 1.000 | 2200.00 |
| **1500 km summed MST distance** | | | | | | | |
| Time * Phase | 4 | -86.3 | 183 | 0.00 | 9.29e-01 | 0.929 | 1.00 |
| Phase Only | 2 | -91.7 | 189 | 6.30 | 3.99e-02 | 0.969 | 23.30 |
| Time + Phase | 3 | -91.0 | 190 | 7.05 | 2.74e-02 | 0.996 | 33.90 |
| Time Only | 2 | -94.4 | 195 | 11.70 | 2.63e-03 | 0.999 | 353.00 |
| Intercept Only | 1 | -96.2 | 196 | 13.30 | 1.22e-03 | 1.000 | 761.00 |
| **2000 km summed MST distance** | | | | | | | |
| Time * Phase | 4 | -90.4 | 191 | 0.00 | 7.99e-01 | 0.799 | 1.00 |
| Time + Phase | 3 | -93.6 | 195 | 4.05 | 1.05e-01 | 0.904 | 7.61 |
| Phase Only | 2 | -95.2 | 197 | 5.26 | 5.76e-02 | 0.962 | 13.90 |
| Intercept Only | 1 | -97.0 | 198 | 6.78 | 2.69e-02 | 0.988 | 29.70 |
| Time Only | 2 | -96.9 | 200 | 8.51 | 1.13e-02 | 1.000 | 70.70 |
| **2500 km summed MST distance** | | | | | | | |
| Time * Phase | 4 | -68.5 | 148 | 0.00 | 9.92e-01 | 0.992 | 1.00 |
| Time + Phase | 3 | -74.9 | 158 | 10.40 | 5.38e-03 | 0.997 | 184.00 |
| Phase Only | 2 | -76.7 | 160 | 12.00 | 2.45e-03 | 1.000 | 405.00 |
| Intercept Only | 1 | -81.5 | 167 | 19.50 | 5.74e-05 | 1.000 | 17300.00 |
| Time Only | 2 | -80.5 | 167 | 19.70 | 5.35e-05 | 1.000 | 18500.00 |
| **3000 km summed MST distance** | | | | | | | |
| Time * Phase | 4 | -59.4 | 129 | 0.00 | 9.45e-01 | 0.945 | 1.00 |
| Time + Phase | 3 | -63.6 | 136 | 6.10 | 4.47e-02 | 0.990 | 21.10 |
| Phase Only | 2 | -66.1 | 138 | 9.00 | 1.05e-02 | 1.000 | 90.00 |
| Time Only | 2 | -71.4 | 149 | 19.50 | 5.45e-05 | 1.000 | 17300.00 |
| Intercept Only | 1 | -73.4 | 151 | 21.40 | 2.12e-05 | 1.000 | 44600.00 |
| **3500 km summed MST distance** | | | | | | | |
| Time * Phase | 4 | -51.6 | 114 | 0.00 | 9.63e-01 | 0.963 | 1.00 |
| Time + Phase | 3 | -56.3 | 121 | 7.21 | 2.61e-02 | 0.989 | 36.90 |
| Phase Only | 2 | -58.3 | 123 | 8.96 | 1.09e-02 | 1.000 | 88.30 |
| Time Only | 2 | -63.5 | 133 | 19.30 | 6.08e-05 | 1.000 | 15800.00 |
| Intercept Only | 1 | -65.6 | 135 | 21.40 | 2.12e-05 | 1.000 | 45400.00 |
| **4000 km summed MST distance** | | | | | | | |
| Time * Phase | 4 | -52.9 | 116 | 0.00 | 9.64e-01 | 0.964 | 1.00 |
| Phase Only | 2 | -59.0 | 124 | 7.81 | 1.94e-02 | 0.983 | 49.70 |
| Time + Phase | 3 | -58.1 | 125 | 8.12 | 1.66e-02 | 1.000 | 58.10 |
| Time Only | 2 | -63.5 | 133 | 16.80 | 2.22e-04 | 1.000 | 4340.00 |
| Intercept Only | 1 | -66.3 | 137 | 20.30 | 3.83e-05 | 1.000 | 25200.00 |

**Table S3.** Coefficients for variables included in generalised least-squares models of ‘global’ species richness (face-value and sampling standardised, using SQS at a quorum of 0.6) as a function of the palaeogeographic spread of the fossil record (counts of occupied equal-area grid cells with 500 km spacings), continental area and non-marine sediment extent (counts of columns in Macrostrat database). Temporally-correlated errors modelled using a first-order autoregressive structure. Palaeogeographic spread and non-marine sediment extent variables log-transformed to achieve normality. When all three explanatory variables are included in a linear model, only palaeogeographic spread (MST distance) is a significant (at p ≤ 0.01) and strong explanation of variation in ‘global’ species richness.

| **term** | **estimate** | **std.error** | **statistic** | **p.value** |
| --- | --- | --- | --- | --- |
| **Face-value Global Species Richness** | | | | |
| Intercept | -2.1600 | 1.8300 | -1.1900 | n.s. |
| Occupied Grid Cells | 0.8340 | 0.0960 | 8.6900 | < 0.01 |
| Non-marine Sediment Extent | -0.0256 | 0.1720 | -0.1490 | n.s. |
| Continental Area | 0.1820 | 0.0785 | 2.3100 | n.s. |
| **SQS Global Richness** | | | | |
| Intercept | 2.4500 | 1.6600 | 1.4700 | n.s. |
| Occupied Grid Cells | 0.5010 | 0.1240 | 4.0400 | < 0.01 |
| Non-marine Sediment Extent | -0.0143 | 0.1880 | -0.0761 | n.s. |
| Continental Area | 0.0413 | 0.0777 | 0.5310 | n.s. |

**Table S4.** Parameter estimates for coefficients in linear models fitted to spatially-standardised terrestrial tetrapod species richness data (SQS, quorum = 0.6; 1000–4000 km MST distance; GCR quota = off). All models fitted to each palaeogeographic spread level are shown, regardless of Akaike weight, and ordering does not reflect importance.

|  | **Intercept** | | | | **Time** | | | | **Time : Phase (Pre-K/Pg)** | | | | **Phase (Pre-K/Pg)** | | | |
| --- | --- | --- | --- | --- | --- | --- | --- | --- | --- | --- | --- | --- | --- | --- | --- | --- |
| **model** | **estimate** | **std.error** | **statistic** | **p.value** | **estimate** | **std.error** | **statistic** | **p.value** | **estimate** | **std.error** | **statistic** | **p.value** | **estimate** | **std.error** | **statistic** | **p.value** |
| **1000 km summed MST distance** | | | | | | | | | | | | | | | | |
| Intercept Only | 3.90 | 0.0534 | 73.1 | < 0.05 |  |  |  |  |  |  |  |  |  |  |  |  |
| Phase Only | 4.02 | 0.0574 | 70.0 | < 0.05 |  |  |  |  |  |  |  |  | -0.471 | 0.114 | -4.12 | < 0.05 |
| Time Only | 4.01 | 0.0636 | 63.0 | < 0.05 | -0.002 | 0.000665 | -3 | < 0.05 |  |  |  |  |  |  |  |  |
| Time + Phase | 4.00 | 0.0617 | 64.9 | < 0.05 | 0.000851 | 0.00121 | 0.705 | n.s. |  |  |  |  | -0.599 | 0.215 | -2.79 | < 0.05 |
| Time * Phase | 3.89 | 0.0764 | 51.0 | < 0.05 | 0.00662 | 0.0028 | 2.37 | < 0.05 | -0.00703 | 0.00309 | -2.27 | < 0.05 | -0.281 | 0.252 | -1.11 | n.s. |
| **1500 km summed MST distance** | | | | | | | | | | | | | | | | |
| Intercept Only | 3.95 | 0.0710 | 55.7 | < 0.05 |  |  |  |  |  |  |  |  |  |  |  |  |
| Phase Only | 4.06 | 0.0762 | 53.3 | < 0.05 |  |  |  |  |  |  |  |  | -0.515 | 0.169 | -3.05 | < 0.05 |
| Time Only | 4.04 | 0.0834 | 48.4 | < 0.05 | -0.00184 | 0.000969 | -1.9 | n.s. |  |  |  |  |  |  |  |  |
| Time + Phase | 4.02 | 0.0810 | 49.7 | < 0.05 | 0.00204 | 0.00176 | 1.16 | n.s. |  |  |  |  | -0.823 | 0.314 | -2.62 | < 0.05 |
| Time * Phase | 3.84 | 0.0981 | 39.2 | < 0.05 | 0.0135 | 0.00412 | 3.28 | < 0.05 | -0.0138 | 0.00452 | -3.05 | < 0.05 | -0.258 | 0.353 | -0.73 | n.s. |
| **2000 km summed MST distance** | | | | | | | | | | | | | | | | |
| Intercept Only | 3.93 | 0.0754 | 52.1 | < 0.05 |  |  |  |  |  |  |  |  |  |  |  |  |
| Phase Only | 4.01 | 0.0861 | 46.6 | < 0.05 |  |  |  |  |  |  |  |  | -0.323 | 0.17 | -1.9 | n.s. |
| Time Only | 3.96 | 0.0938 | 42.2 | < 0.05 | -0.000599 | 0.001 | -0.596 | n.s. |  |  |  |  |  |  |  |  |
| Time + Phase | 3.95 | 0.0910 | 43.4 | < 0.05 | 0.00323 | 0.00178 | 1.82 | n.s. |  |  |  |  | -0.791 | 0.307 | -2.57 | < 0.05 |
| Time * Phase | 3.78 | 0.1130 | 33.4 | < 0.05 | 0.0128 | 0.00423 | 3.03 | < 0.05 | -0.0115 | 0.00464 | -2.48 | < 0.05 | -0.302 | 0.357 | -0.846 | n.s. |
| **2500 km summed MST distance** | | | | | | | | | | | | | | | | |
| Intercept Only | 3.98 | 0.0655 | 60.8 | < 0.05 |  |  |  |  |  |  |  |  |  |  |  |  |
| Phase Only | 4.11 | 0.0743 | 55.3 | < 0.05 |  |  |  |  |  |  |  |  | -0.431 | 0.137 | -3.15 | < 0.05 |
| Time Only | 4.05 | 0.0802 | 50.5 | < 0.05 | -0.00111 | 0.000798 | -1.39 | n.s. |  |  |  |  |  |  |  |  |
| Time + Phase | 4.07 | 0.0759 | 53.6 | < 0.05 | 0.00247 | 0.00129 | 1.92 | n.s. |  |  |  |  | -0.789 | 0.23 | -3.42 | < 0.05 |
| Time * Phase | 3.86 | 0.0915 | 42.2 | < 0.05 | 0.0158 | 0.00389 | 4.06 | < 0.05 | -0.0147 | 0.00409 | -3.6 | < 0.05 | -0.354 | 0.247 | -1.43 | n.s. |
| **3000 km summed MST distance** | | | | | | | | | | | | | | | | |
| Intercept Only | 4.05 | 0.0707 | 57.2 | < 0.05 |  |  |  |  |  |  |  |  |  |  |  |  |
| Phase Only | 4.20 | 0.0758 | 55.4 | < 0.05 |  |  |  |  |  |  |  |  | -0.576 | 0.146 | -3.94 | < 0.05 |
| Time Only | 4.14 | 0.0851 | 48.7 | < 0.05 | -0.00174 | 0.00087 | -2 | < 0.05 |  |  |  |  |  |  |  |  |
| Time + Phase | 4.15 | 0.0775 | 53.5 | < 0.05 | 0.00329 | 0.00147 | 2.24 | < 0.05 |  |  |  |  | -1.07 | 0.264 | -4.07 | < 0.05 |
| Time * Phase | 3.98 | 0.0946 | 42.0 | < 0.05 | 0.014 | 0.00396 | 3.53 | < 0.05 | -0.0122 | 0.00423 | -2.89 | < 0.05 | -0.648 | 0.292 | -2.22 | < 0.05 |
| **3500 km summed MST distance** | | | | | | | | | | | | | | | | |
| Intercept Only | 4.13 | 0.0650 | 63.5 | < 0.05 |  |  |  |  |  |  |  |  |  |  |  |  |
| Phase Only | 4.29 | 0.0718 | 59.7 | < 0.05 |  |  |  |  |  |  |  |  | -0.509 | 0.129 | -3.96 | < 0.05 |
| Time Only | 4.22 | 0.0795 | 53.1 | < 0.05 | -0.00151 | 0.000736 | -2.06 | < 0.05 |  |  |  |  |  |  |  |  |
| Time + Phase | 4.25 | 0.0732 | 58.0 | < 0.05 | 0.00237 | 0.00121 | 1.96 | n.s. |  |  |  |  | -0.876 | 0.225 | -3.88 | < 0.05 |
| Time * Phase | 4.07 | 0.0895 | 45.5 | < 0.05 | 0.013 | 0.00364 | 3.58 | < 0.05 | -0.0118 | 0.00384 | -3.09 | < 0.05 | -0.501 | 0.245 | -2.04 | < 0.05 |
| **4000 km summed MST distance** | | | | | | | | | | | | | | | | |
| Intercept Only | 4.17 | 0.0751 | 55.5 | < 0.05 |  |  |  |  |  |  |  |  |  |  |  |  |
| Phase Only | 4.35 | 0.0824 | 52.8 | < 0.05 |  |  |  |  |  |  |  |  | -0.583 | 0.147 | -3.97 | < 0.05 |
| Time Only | 4.30 | 0.0912 | 47.2 | < 0.05 | -0.00197 | 0.000824 | -2.39 | < 0.05 |  |  |  |  |  |  |  |  |
| Time + Phase | 4.32 | 0.0852 | 50.7 | < 0.05 | 0.00186 | 0.00138 | 1.35 | n.s. |  |  |  |  | -0.877 | 0.262 | -3.35 | < 0.05 |
| Time * Phase | 4.10 | 0.1060 | 38.8 | < 0.05 | 0.015 | 0.00425 | 3.53 | < 0.05 | -0.0145 | 0.00446 | -3.25 | < 0.05 | -0.418 | 0.283 | -1.48 | n.s. |

**Table S5.** Model selection using the second-order Akaike information criterion (AICc) to compare fits of linear models of spatially-standardised non-flying terrestrial species richness (SQS, quorum = 0.6; GCR quota = 5 occupied grid cells/1000 km MST length) as a function of time and diversification phase.

| **model** | **df** | **logLik** | **AICc** | **delta AICc** | **weights** | **cumulative weights** | **evidence ratio** |
| --- | --- | --- | --- | --- | --- | --- | --- |
| **1000 km summed MST distance** | | | | | | | |
| Time * Phase | 4 | -69.7 | 150.0 | 0.00 | 8.76e-01 | 0.876 | 1.00 |
| Phase Only | 2 | -74.3 | 155.0 | 4.82 | 7.88e-02 | 0.955 | 11.10 |
| Time + Phase | 3 | -73.8 | 156.0 | 5.97 | 4.44e-02 | 0.999 | 19.70 |
| Time Only | 2 | -79.0 | 164.0 | 14.20 | 7.08e-04 | 1.000 | 1240.00 |
| Intercept Only | 1 | -84.0 | 172.0 | 22.10 | 1.40e-05 | 1.000 | 62600.00 |
| **1500 km summed MST distance** | | | | | | | |
| Time * Phase | 4 | -82.4 | 175.0 | 0.00 | 9.49e-01 | 0.949 | 1.00 |
| Phase Only | 2 | -88.0 | 182.0 | 6.95 | 2.94e-02 | 0.978 | 32.30 |
| Time + Phase | 3 | -87.5 | 183.0 | 8.00 | 1.74e-02 | 0.996 | 54.50 |
| Time Only | 2 | -90.3 | 187.0 | 11.60 | 2.89e-03 | 0.999 | 328.00 |
| Intercept Only | 1 | -92.2 | 188.0 | 13.30 | 1.24e-03 | 1.000 | 765.00 |
| **2000 km summed MST distance** | | | | | | | |
| Time * Phase | 4 | -79.8 | 170.0 | 0.00 | 9.67e-01 | 0.967 | 1.00 |
| Time + Phase | 3 | -85.0 | 178.0 | 8.17 | 1.63e-02 | 0.983 | 59.30 |
| Phase Only | 2 | -86.4 | 179.0 | 8.86 | 1.15e-02 | 0.995 | 84.10 |
| Intercept Only | 1 | -88.7 | 181.0 | 11.30 | 3.41e-03 | 0.998 | 284.00 |
| Time Only | 2 | -88.3 | 183.0 | 12.60 | 1.78e-03 | 1.000 | 543.00 |
| **2500 km summed MST distance** | | | | | | | |
| Time * Phase | 4 | -56.2 | 123.0 | 0.00 | 9.98e-01 | 0.998 | 1.00 |
| Phase Only | 2 | -65.5 | 137.0 | 14.20 | 8.16e-04 | 0.999 | 1220.00 |
| Time + Phase | 3 | -64.6 | 137.0 | 14.50 | 7.00e-04 | 1.000 | 1430.00 |
| Intercept Only | 1 | -67.4 | 139.0 | 15.90 | 3.47e-04 | 1.000 | 2880.00 |
| Time Only | 2 | -67.1 | 140.0 | 17.30 | 1.71e-04 | 1.000 | 5840.00 |
| **3000 km summed MST distance** | | | | | | | |
| Time * Phase | 4 | -42.3 | 95.3 | 0.00 | 9.95e-01 | 0.995 | 1.00 |
| Time + Phase | 3 | -49.3 | 107.0 | 11.60 | 3.03e-03 | 0.998 | 328.00 |
| Phase Only | 2 | -50.9 | 108.0 | 12.60 | 1.81e-03 | 1.000 | 550.00 |
| Time Only | 2 | -54.8 | 116.0 | 20.50 | 3.45e-05 | 1.000 | 28800.00 |
| Intercept Only | 1 | -56.5 | 117.0 | 21.80 | 1.87e-05 | 1.000 | 53200.00 |
| **3500 km summed MST distance** | | | | | | | |
| Time * Phase | 4 | -35.6 | 82.0 | 0.00 | 9.69e-01 | 0.969 | 1.00 |
| Time + Phase | 3 | -40.8 | 90.1 | 8.13 | 1.67e-02 | 0.986 | 58.00 |
| Phase Only | 2 | -42.2 | 90.7 | 8.72 | 1.24e-02 | 0.998 | 78.10 |
| Time Only | 2 | -45.0 | 96.2 | 14.20 | 7.91e-04 | 0.999 | 1230.00 |
| Intercept Only | 1 | -46.1 | 96.4 | 14.40 | 7.32e-04 | 1.000 | 1320.00 |
| **4000 km summed MST distance** | | | | | | | |
| Time * Phase | 4 | -34.2 | 79.3 | 0.00 | 8.46e-01 | 0.846 | 1.00 |
| Time + Phase | 3 | -37.6 | 83.8 | 4.47 | 9.04e-02 | 0.936 | 9.36 |
| Phase Only | 2 | -39.3 | 84.8 | 5.47 | 5.48e-02 | 0.991 | 15.40 |
| Intercept Only | 1 | -42.8 | 89.7 | 10.40 | 4.73e-03 | 0.996 | 179.00 |
| Time Only | 2 | -41.8 | 89.9 | 10.60 | 4.24e-03 | 1.000 | 200.00 |

**Table S6.** Parameter estimates for coefficients in linear models fitted to spatially-standardised terrestrial tetrapod species richness data (SQS, quorum = 0.6; GCR quota = 5 occupied grid-cells/1000 km MST length). All models fitted to each palaeogeographic spread level are shown, regardless of Akaike weight, and ordering does not reflect importance.

|  | **Intercept** | | | | **Time** | | | | **Time : Phase (Pre-K/Pg)** | | | | **Phase (Pre-K/Pg)** | | | |
| --- | --- | --- | --- | --- | --- | --- | --- | --- | --- | --- | --- | --- | --- | --- | --- | --- |
| **model** | **estimate** | **std.error** | **statistic** | **p.value** | **estimate** | **std.error** | **statistic** | **p.value** | **estimate** | **std.error** | **statistic** | **p.value** | **estimate** | **std.error** | **statistic** | **p.value** |
| **1000 km summed MST distance** | | | | | | | | | | | | | | | | |
| Intercept Only | 3.70 | 0.0579 | 64.0 | < 0.05 |  |  |  |  |  |  |  |  |  |  |  |  |
| Phase Only | 3.85 | 0.0615 | 62.6 | < 0.05 |  |  |  |  |  |  |  |  | -0.546 | 0.119 | -4.58 | < 0.05 |
| Time Only | 3.83 | 0.0685 | 55.9 | < 0.05 | -0.00224 | 0.000701 | -3.2 | < 0.05 |  |  |  |  |  |  |  |  |
| Time + Phase | 3.82 | 0.0653 | 58.5 | < 0.05 | 0.00123 | 0.00126 | 0.977 | n.s. |  |  |  |  | -0.732 | 0.224 | -3.26 | < 0.05 |
| Time * Phase | 3.69 | 0.0790 | 46.7 | < 0.05 | 0.00898 | 0.00297 | 3.02 | < 0.05 | -0.00929 | 0.00326 | -2.85 | < 0.05 | -0.335 | 0.257 | -1.3 | n.s. |
| **1500 km summed MST distance** | | | | | | | | | | | | | | | | |
| Intercept Only | 3.80 | 0.0703 | 54.1 | < 0.05 |  |  |  |  |  |  |  |  |  |  |  |  |
| Phase Only | 3.89 | 0.0748 | 52.0 | < 0.05 |  |  |  |  |  |  |  |  | -0.509 | 0.173 | -2.94 | < 0.05 |
| Time Only | 3.88 | 0.0818 | 47.5 | < 0.05 | -0.00185 | 0.000952 | -1.94 | n.s. |  |  |  |  |  |  |  |  |
| Time + Phase | 3.86 | 0.0802 | 48.2 | < 0.05 | 0.00186 | 0.00181 | 1.03 | n.s. |  |  |  |  | -0.807 | 0.337 | -2.39 | < 0.05 |
| Time * Phase | 3.68 | 0.0959 | 38.4 | < 0.05 | 0.0136 | 0.00403 | 3.37 | < 0.05 | -0.0143 | 0.00446 | -3.21 | < 0.05 | -0.161 | 0.379 | -0.425 | n.s. |
| **2000 km summed MST distance** | | | | | | | | | | | | | | | | |
| Intercept Only | 3.87 | 0.0825 | 47.0 | < 0.05 |  |  |  |  |  |  |  |  |  |  |  |  |
| Phase Only | 3.98 | 0.0948 | 42.0 | < 0.05 |  |  |  |  |  |  |  |  | -0.386 | 0.181 | -2.13 | < 0.05 |
| Time Only | 3.93 | 0.1020 | 38.5 | < 0.05 | -0.000934 | 0.00105 | -0.892 | n.s. |  |  |  |  |  |  |  |  |
| Time + Phase | 3.93 | 0.0986 | 39.8 | < 0.05 | 0.00315 | 0.00188 | 1.67 | n.s. |  |  |  |  | -0.856 | 0.333 | -2.57 | < 0.05 |
| Time * Phase | 3.69 | 0.1190 | 30.9 | < 0.05 | 0.0179 | 0.00489 | 3.67 | < 0.05 | -0.017 | 0.00525 | -3.25 | < 0.05 | -0.241 | 0.367 | -0.658 | n.s. |
| **2500 km summed MST distance** | | | | | | | | | | | | | | | | |
| Intercept Only | 3.97 | 0.0666 | 59.6 | < 0.05 |  |  |  |  |  |  |  |  |  |  |  |  |
| Phase Only | 4.04 | 0.0741 | 54.5 | < 0.05 |  |  |  |  |  |  |  |  | -0.308 | 0.158 | -1.95 | n.s. |
| Time Only | 4.01 | 0.0800 | 50.1 | < 0.05 | -0.00077 | 0.000936 | -0.823 | n.s. |  |  |  |  |  |  |  |  |
| Time + Phase | 4.00 | 0.0780 | 51.3 | < 0.05 | 0.00215 | 0.0016 | 1.35 | n.s. |  |  |  |  | -0.612 | 0.275 | -2.23 | < 0.05 |
| Time * Phase | 3.77 | 0.0894 | 42.2 | < 0.05 | 0.0167 | 0.00374 | 4.46 | < 0.05 | -0.0171 | 0.00406 | -4.21 | < 0.05 | 0.0199 | 0.29 | 0.0685 | n.s. |
| **3000 km summed MST distance** | | | | | | | | | | | | | | | | |
| Intercept Only | 4.02 | 0.0737 | 54.5 | < 0.05 |  |  |  |  |  |  |  |  |  |  |  |  |
| Phase Only | 4.15 | 0.0778 | 53.3 | < 0.05 |  |  |  |  |  |  |  |  | -0.555 | 0.161 | -3.45 | < 0.05 |
| Time Only | 4.11 | 0.0873 | 47.0 | < 0.05 | -0.00173 | 0.00095 | -1.83 | n.s. |  |  |  |  |  |  |  |  |
| Time + Phase | 4.10 | 0.0807 | 50.8 | < 0.05 | 0.00286 | 0.00161 | 1.78 | n.s. |  |  |  |  | -0.987 | 0.29 | -3.41 | < 0.05 |
| Time * Phase | 3.89 | 0.0923 | 42.1 | < 0.05 | 0.0163 | 0.00382 | 4.27 | < 0.05 | -0.0158 | 0.00413 | -3.81 | < 0.05 | -0.39 | 0.305 | -1.28 | n.s. |
| **3500 km summed MST distance** | | | | | | | | | | | | | | | | |
| Intercept Only | 4.18 | 0.0695 | 60.2 | < 0.05 |  |  |  |  |  |  |  |  |  |  |  |  |
| Phase Only | 4.28 | 0.0743 | 57.6 | < 0.05 |  |  |  |  |  |  |  |  | -0.449 | 0.158 | -2.84 | < 0.05 |
| Time Only | 4.25 | 0.0821 | 51.8 | < 0.05 | -0.00129 | 0.000857 | -1.51 | n.s. |  |  |  |  |  |  |  |  |
| Time + Phase | 4.24 | 0.0772 | 54.9 | < 0.05 | 0.00257 | 0.00155 | 1.66 | n.s. |  |  |  |  | -0.873 | 0.3 | -2.91 | < 0.05 |
| Time * Phase | 4.07 | 0.0883 | 46.1 | < 0.05 | 0.0132 | 0.00357 | 3.71 | < 0.05 | -0.0127 | 0.0039 | -3.26 | < 0.05 | -0.333 | 0.323 | -1.03 | n.s. |
| **4000 km summed MST distance** | | | | | | | | | | | | | | | | |
| Intercept Only | 4.17 | 0.0792 | 52.7 | < 0.05 |  |  |  |  |  |  |  |  |  |  |  |  |
| Phase Only | 4.30 | 0.0876 | 49.0 | < 0.05 |  |  |  |  |  |  |  |  | -0.451 | 0.167 | -2.7 | < 0.05 |
| Time Only | 4.25 | 0.0959 | 44.3 | < 0.05 | -0.00124 | 0.000899 | -1.38 | n.s. |  |  |  |  |  |  |  |  |
| Time + Phase | 4.25 | 0.0893 | 47.7 | < 0.05 | 0.00296 | 0.00166 | 1.78 | n.s. |  |  |  |  | -0.951 | 0.325 | -2.93 | < 0.05 |
| Time * Phase | 4.09 | 0.1060 | 38.4 | < 0.05 | 0.0142 | 0.00462 | 3.08 | < 0.05 | -0.0128 | 0.00491 | -2.6 | < 0.05 | -0.513 | 0.35 | -1.46 | n.s. |

**Table S7.** Model selection using the second-order Akaike information criterion (AICc) to compare fits of linear models of spatially-standardised non-flying terrestrial species richness (face-value species counts) as a function of time and diversification phase.

| **model** | **df** | **logLik** | **AICc** | **delta AICc** | **weights** | **cumulative weights** | **evidence ratio** |
| --- | --- | --- | --- | --- | --- | --- | --- |
| **1000 km summed MST distance** | | | | | | | |
| Time * Phase | 4 | -76.2 | 163 | 0.00 | 8.20e-01 | 0.820 | 1.00e+00 |
| Phase Only | 2 | -80.2 | 166 | 3.65 | 1.32e-01 | 0.952 | 6.21e+00 |
| Time + Phase | 3 | -80.2 | 169 | 5.77 | 4.57e-02 | 0.998 | 1.79e+01 |
| Time Only | 2 | -84.1 | 174 | 11.60 | 2.52e-03 | 1.000 | 3.25e+02 |
| Intercept Only | 1 | -93.0 | 190 | 27.20 | 1.00e-06 | 1.000 | 8.12e+05 |
| **1500 km summed MST distance** | | | | | | | |
| Time * Phase | 4 | -78.9 | 168 | 0.00 | 9.81e-01 | 0.981 | 1.00e+00 |
| Phase Only | 2 | -85.4 | 177 | 8.69 | 1.28e-02 | 0.994 | 7.66e+01 |
| Time + Phase | 3 | -85.2 | 179 | 10.50 | 5.20e-03 | 0.999 | 1.89e+02 |
| Time Only | 2 | -88.4 | 183 | 14.80 | 5.90e-04 | 1.000 | 1.66e+03 |
| Intercept Only | 1 | -92.5 | 189 | 20.80 | 2.99e-05 | 1.000 | 3.28e+04 |
| **2000 km summed MST distance** | | | | | | | |
| Time * Phase | 4 | -79.5 | 169 | 0.00 | 8.34e-01 | 0.834 | 1.00e+00 |
| Phase Only | 2 | -83.7 | 174 | 4.23 | 1.00e-01 | 0.934 | 8.34e+00 |
| Time + Phase | 3 | -83.2 | 175 | 5.22 | 6.15e-02 | 0.995 | 1.36e+01 |
| Time Only | 2 | -87.1 | 180 | 10.90 | 3.50e-03 | 0.999 | 2.38e+02 |
| Intercept Only | 1 | -89.7 | 183 | 14.00 | 7.49e-04 | 1.000 | 1.11e+03 |
| **2500 km summed MST distance** | | | | | | | |
| Time * Phase | 4 | -79.5 | 169 | 0.00 | 9.73e-01 | 0.973 | 1.00e+00 |
| Phase Only | 2 | -85.5 | 177 | 7.79 | 1.98e-02 | 0.993 | 4.91e+01 |
| Time + Phase | 3 | -85.5 | 179 | 9.85 | 7.07e-03 | 1.000 | 1.38e+02 |
| Time Only | 2 | -90.0 | 186 | 16.70 | 2.32e-04 | 1.000 | 4.19e+03 |
| Intercept Only | 1 | -95.9 | 196 | 26.50 | 1.70e-06 | 1.000 | 5.66e+05 |
| **3000 km summed MST distance** | | | | | | | |
| Time * Phase | 4 | -70.4 | 151 | 0.00 | 9.77e-01 | 0.977 | 1.00e+00 |
| Phase Only | 2 | -76.7 | 159 | 8.12 | 1.68e-02 | 0.994 | 5.82e+01 |
| Time + Phase | 3 | -76.6 | 162 | 10.20 | 5.88e-03 | 1.000 | 1.66e+02 |
| Time Only | 2 | -80.1 | 166 | 14.90 | 5.54e-04 | 1.000 | 1.76e+03 |
| Intercept Only | 1 | -85.9 | 176 | 24.60 | 4.40e-06 | 1.000 | 2.20e+05 |
| **3500 km summed MST distance** | | | | | | | |
| Time * Phase | 4 | -68.5 | 147 | 0.00 | 9.56e-01 | 0.956 | 1.00e+00 |
| Phase Only | 2 | -74.1 | 154 | 6.91 | 3.02e-02 | 0.986 | 3.17e+01 |
| Time + Phase | 3 | -73.8 | 156 | 8.45 | 1.40e-02 | 1.000 | 6.83e+01 |
| Time Only | 2 | -80.3 | 167 | 19.20 | 6.41e-05 | 1.000 | 1.49e+04 |
| Intercept Only | 1 | -86.9 | 178 | 30.40 | 2.00e-07 | 1.000 | 4.00e+06 |
| **4000 km summed MST distance** | | | | | | | |
| Time * Phase | 4 | -68.1 | 147 | 0.00 | 8.84e-01 | 0.884 | 1.00e+00 |
| Phase Only | 2 | -72.6 | 151 | 4.74 | 8.28e-02 | 0.967 | 1.07e+01 |
| Time + Phase | 3 | -72.5 | 153 | 6.65 | 3.18e-02 | 0.999 | 2.78e+01 |
| Time Only | 2 | -76.8 | 160 | 13.10 | 1.26e-03 | 1.000 | 7.02e+02 |
| Intercept Only | 1 | -82.1 | 168 | 21.50 | 1.89e-05 | 1.000 | 4.68e+04 |

**Table S8.** Parameter estimates for coefficients in linear models fitted to spatially-standardised terrestrial tetrapod species richness data (face-value species counts). All models fitted to each palaeogeographic spread level are shown, regardless of Akaike weight, and ordering does not reflect importance.

|  | **Intercept** | | | | **Time** | | | | **Time : Phase (Pre-K/Pg)** | | | | **Phase (Pre-K/Pg)** | | | |
| --- | --- | --- | --- | --- | --- | --- | --- | --- | --- | --- | --- | --- | --- | --- | --- | --- |
| **model** | **estimate** | **std.error** | **statistic** | **p.value** | **estimate** | **std.error** | **statistic** | **p.value** | **estimate** | **std.error** | **statistic** | **p.value** | **estimate** | **std.error** | **statistic** | **p.value** |
| **1000 km summed MST distance** | | | | | | | | | | | | | | | | |
| Intercept Only | 4.44 | 0.0591 | 75.2 | < 0.05 |  |  |  |  |  |  |  |  |  |  |  |  |
| Phase Only | 4.61 | 0.0607 | 75.9 | < 0.05 |  |  |  |  |  |  |  |  | -0.645 | 0.121 | -5.34 | < 0.05 |
| Time Only | 4.62 | 0.0675 | 68.4 | < 0.05 | -0.00307 | 0.000706 | -4.35 | < 0.05 |  |  |  |  |  |  |  |  |
| Time + Phase | 4.61 | 0.0654 | 70.5 | < 0.05 | -1.59e-05 | 0.00128 | -0.0124 | n.s. |  |  |  |  | -0.643 | 0.227 | -2.83 | < 0.05 |
| Time * Phase | 4.47 | 0.0799 | 55.9 | < 0.05 | 0.00746 | 0.00293 | 2.55 | < 0.05 | -0.0091 | 0.00323 | -2.82 | < 0.05 | -0.231 | 0.264 | -0.875 | n.s. |
| **1500 km summed MST distance** | | | | | | | | | | | | | | | | |
| Intercept Only | 4.56 | 0.0682 | 66.8 | < 0.05 |  |  |  |  |  |  |  |  |  |  |  |  |
| Phase Only | 4.68 | 0.0712 | 65.7 | < 0.05 |  |  |  |  |  |  |  |  | -0.61 | 0.158 | -3.87 | < 0.05 |
| Time Only | 4.68 | 0.0783 | 59.8 | < 0.05 | -0.00261 | 0.000909 | -2.87 | < 0.05 |  |  |  |  |  |  |  |  |
| Time + Phase | 4.67 | 0.0762 | 61.3 | < 0.05 | 0.000948 | 0.00165 | 0.575 | n.s. |  |  |  |  | -0.754 | 0.296 | -2.55 | < 0.05 |
| Time * Phase | 4.47 | 0.0905 | 49.3 | < 0.05 | 0.0135 | 0.00381 | 3.54 | < 0.05 | -0.015 | 0.00417 | -3.6 | < 0.05 | -0.137 | 0.326 | -0.421 | n.s. |
| **2000 km summed MST distance** | | | | | | | | | | | | | | | | |
| Intercept Only | 4.55 | 0.0695 | 65.4 | < 0.05 |  |  |  |  |  |  |  |  |  |  |  |  |
| Phase Only | 4.68 | 0.0758 | 61.8 | < 0.05 |  |  |  |  |  |  |  |  | -0.529 | 0.15 | -3.53 | < 0.05 |
| Time Only | 4.66 | 0.0842 | 55.4 | < 0.05 | -0.00206 | 0.000901 | -2.28 | < 0.05 |  |  |  |  |  |  |  |  |
| Time + Phase | 4.65 | 0.0811 | 57.3 | < 0.05 | 0.00168 | 0.00158 | 1.06 | n.s. |  |  |  |  | -0.772 | 0.274 | -2.82 | < 0.05 |
| Time * Phase | 4.48 | 0.1000 | 44.7 | < 0.05 | 0.011 | 0.00375 | 2.93 | < 0.05 | -0.0111 | 0.0041 | -2.72 | < 0.05 | -0.3 | 0.316 | -0.947 | n.s. |
| **2500 km summed MST distance** | | | | | | | | | | | | | | | | |
| Intercept Only | 4.51 | 0.0772 | 58.4 | < 0.05 |  |  |  |  |  |  |  |  |  |  |  |  |
| Phase Only | 4.72 | 0.0821 | 57.5 | < 0.05 |  |  |  |  |  |  |  |  | -0.724 | 0.151 | -4.79 | < 0.05 |
| Time Only | 4.69 | 0.0892 | 52.6 | < 0.05 | -0.00313 | 0.000888 | -3.53 | < 0.05 |  |  |  |  |  |  |  |  |
| Time + Phase | 4.71 | 0.0856 | 55.0 | < 0.05 | 0.000427 | 0.00145 | 0.294 | n.s. |  |  |  |  | -0.785 | 0.26 | -3.02 | < 0.05 |
| Time * Phase | 4.48 | 0.1040 | 43.3 | < 0.05 | 0.0151 | 0.00441 | 3.43 | < 0.05 | -0.0163 | 0.00463 | -3.51 | < 0.05 | -0.305 | 0.28 | -1.09 | n.s. |
| **3000 km summed MST distance** | | | | | | | | | | | | | | | | |
| Intercept Only | 4.67 | 0.0830 | 56.3 | < 0.05 |  |  |  |  |  |  |  |  |  |  |  |  |
| Phase Only | 4.88 | 0.0867 | 56.2 | < 0.05 |  |  |  |  |  |  |  |  | -0.756 | 0.167 | -4.52 | < 0.05 |
| Time Only | 4.87 | 0.0951 | 51.2 | < 0.05 | -0.00342 | 0.000972 | -3.52 | < 0.05 |  |  |  |  |  |  |  |  |
| Time + Phase | 4.87 | 0.0916 | 53.2 | < 0.05 | 0.000422 | 0.00173 | 0.243 | n.s. |  |  |  |  | -0.82 | 0.312 | -2.63 | < 0.05 |
| Time * Phase | 4.63 | 0.1090 | 42.5 | < 0.05 | 0.0157 | 0.00456 | 3.44 | < 0.05 | -0.0174 | 0.00487 | -3.58 | < 0.05 | -0.212 | 0.336 | -0.632 | n.s. |
| **3500 km summed MST distance** | | | | | | | | | | | | | | | | |
| Intercept Only | 4.72 | 0.0858 | 55.0 | < 0.05 |  |  |  |  |  |  |  |  |  |  |  |  |
| Phase Only | 4.99 | 0.0882 | 56.5 | < 0.05 |  |  |  |  |  |  |  |  | -0.859 | 0.158 | -5.44 | < 0.05 |
| Time Only | 4.94 | 0.0989 | 50.0 | < 0.05 | -0.00344 | 0.000915 | -3.76 | < 0.05 |  |  |  |  |  |  |  |  |
| Time + Phase | 4.97 | 0.0918 | 54.1 | < 0.05 | 0.00117 | 0.00151 | 0.775 | n.s. |  |  |  |  | -1.04 | 0.283 | -3.68 | < 0.05 |
| Time * Phase | 4.73 | 0.1110 | 42.5 | < 0.05 | 0.0154 | 0.00454 | 3.39 | < 0.05 | -0.0157 | 0.00478 | -3.3 | < 0.05 | -0.543 | 0.306 | -1.78 | n.s. |
| **4000 km summed MST distance** | | | | | | | | | | | | | | | | |
| Intercept Only | 4.75 | 0.0941 | 50.5 | < 0.05 |  |  |  |  |  |  |  |  |  |  |  |  |
| Phase Only | 5.01 | 0.1000 | 50.1 | < 0.05 |  |  |  |  |  |  |  |  | -0.819 | 0.178 | -4.59 | < 0.05 |
| Time Only | 4.97 | 0.1100 | 45.1 | < 0.05 | -0.00331 | 0.000997 | -3.32 | < 0.05 |  |  |  |  |  |  |  |  |
| Time + Phase | 5.00 | 0.1050 | 47.7 | < 0.05 | 0.000864 | 0.00169 | 0.51 | n.s. |  |  |  |  | -0.955 | 0.322 | -2.97 | < 0.05 |
| Time * Phase | 4.74 | 0.1310 | 36.2 | < 0.05 | 0.0159 | 0.00527 | 3.01 | < 0.05 | -0.0166 | 0.00553 | -2.99 | < 0.05 | -0.431 | 0.351 | -1.23 | n.s. |

**Table S9.** Model selection using the second-order Akaike information criterion (AICc) to compare fits of linear models of spatially-standardised non-flying terrestrial species richness (squares extrapolated species richness) as a function of time and diversification phase.

| **model** | **df** | **logLik** | **AICc** | **delta AICc** | **weights** | **cumulative weights** | **evidence ratio** |
| --- | --- | --- | --- | --- | --- | --- | --- |
| **1000 km summed MST distance** | | | | | | | |
| Time * Phase | 4 | -74.3 | 159 | 0.00 | 9.73e-01 | 0.973 | 1.00e+00 |
| Phase Only | 2 | -80.6 | 167 | 8.43 | 1.44e-02 | 0.987 | 6.76e+01 |
| Time + Phase | 3 | -79.7 | 168 | 8.75 | 1.22e-02 | 1.000 | 7.98e+01 |
| Time Only | 2 | -85.1 | 176 | 17.40 | 1.65e-04 | 1.000 | 5.90e+03 |
| Intercept Only | 1 | -88.5 | 181 | 22.20 | 1.50e-05 | 1.000 | 6.49e+04 |
| **1500 km summed MST distance** | | | | | | | |
| Time * Phase | 4 | -77.1 | 165 | 0.00 | 9.82e-01 | 0.982 | 1.00e+00 |
| Phase Only | 2 | -84.0 | 174 | 9.35 | 9.16e-03 | 0.991 | 1.07e+02 |
| Time + Phase | 3 | -83.1 | 174 | 9.67 | 7.81e-03 | 0.999 | 1.26e+02 |
| Time Only | 2 | -86.9 | 180 | 15.30 | 4.76e-04 | 0.999 | 2.06e+03 |
| Intercept Only | 1 | -88.6 | 181 | 16.60 | 2.41e-04 | 1.000 | 4.07e+03 |
| **2000 km summed MST distance** | | | | | | | |
| Time * Phase | 4 | -76.8 | 164 | 0.00 | 8.65e-01 | 0.865 | 1.00e+00 |
| Time + Phase | 3 | -80.3 | 169 | 4.88 | 7.54e-02 | 0.940 | 1.15e+01 |
| Phase Only | 2 | -81.9 | 170 | 5.95 | 4.42e-02 | 0.985 | 1.96e+01 |
| Intercept Only | 1 | -84.4 | 173 | 8.85 | 1.04e-02 | 0.995 | 8.32e+01 |
| Time Only | 2 | -84.0 | 174 | 10.10 | 5.46e-03 | 1.000 | 1.58e+02 |
| **2500 km summed MST distance** | | | | | | | |
| Time * Phase | 4 | -75.0 | 160 | 0.00 | 9.95e-01 | 0.995 | 1.00e+00 |
| Phase Only | 2 | -82.9 | 172 | 11.50 | 3.21e-03 | 0.998 | 3.10e+02 |
| Time + Phase | 3 | -82.5 | 173 | 12.70 | 1.70e-03 | 1.000 | 5.85e+02 |
| Time Only | 2 | -87.0 | 180 | 19.80 | 5.09e-05 | 1.000 | 1.95e+04 |
| Intercept Only | 1 | -90.2 | 184 | 24.00 | 6.00e-06 | 1.000 | 1.66e+05 |
| **3000 km summed MST distance** | | | | | | | |
| Time * Phase | 4 | -67.1 | 145 | 0.00 | 9.95e-01 | 0.995 | 1.00e+00 |
| Phase Only | 2 | -75.0 | 156 | 11.30 | 3.50e-03 | 0.998 | 2.84e+02 |
| Time + Phase | 3 | -74.8 | 158 | 13.10 | 1.45e-03 | 1.000 | 6.86e+02 |
| Time Only | 2 | -77.5 | 161 | 16.30 | 2.85e-04 | 1.000 | 3.49e+03 |
| Intercept Only | 1 | -80.3 | 165 | 19.90 | 4.66e-05 | 1.000 | 2.14e+04 |
| **3500 km summed MST distance** | | | | | | | |
| Time * Phase | 4 | -61.8 | 134 | 0.00 | 9.92e-01 | 0.992 | 1.00e+00 |
| Time + Phase | 3 | -68.4 | 145 | 11.10 | 3.87e-03 | 0.996 | 2.56e+02 |
| Phase Only | 2 | -69.5 | 145 | 11.10 | 3.86e-03 | 1.000 | 2.57e+02 |
| Time Only | 2 | -75.2 | 156 | 22.40 | 1.35e-05 | 1.000 | 7.35e+04 |
| Intercept Only | 1 | -78.8 | 162 | 27.60 | 1.00e-06 | 1.000 | 1.01e+06 |
| **4000 km summed MST distance** | | | | | | | |
| Time * Phase | 4 | -64.2 | 139 | 0.00 | 9.49e-01 | 0.949 | 1.00e+00 |
| Phase Only | 2 | -69.8 | 146 | 6.80 | 3.17e-02 | 0.981 | 2.99e+01 |
| Time + Phase | 3 | -69.3 | 147 | 7.94 | 1.79e-02 | 0.999 | 5.30e+01 |
| Time Only | 2 | -73.2 | 152 | 13.50 | 1.10e-03 | 1.000 | 8.63e+02 |
| Intercept Only | 1 | -75.7 | 155 | 16.50 | 2.53e-04 | 1.000 | 3.75e+03 |

**Table S10.** Parameter estimates for coefficients in linear models fitted to spatially-standardised terrestrial tetrapod species richness data (squares extrapolated species richness). All models fitted to each palaeogeographic spread level are shown, regardless of Akaike weight, and ordering does not reflect importance.

|  | **Intercept** | | | | **Time** | | | | **Time : Phase (Pre-K/Pg)** | | | | **Phase (Pre-K/Pg)** | | | |
| --- | --- | --- | --- | --- | --- | --- | --- | --- | --- | --- | --- | --- | --- | --- | --- | --- |
| **model** | **estimate** | **std.error** | **statistic** | **p.value** | **estimate** | **std.error** | **statistic** | **p.value** | **estimate** | **std.error** | **statistic** | **p.value** | **estimate** | **std.error** | **statistic** | **p.value** |
| **1000 km summed MST distance** | | | | | | | | | | | | | | | | |
| Intercept Only | 4.97 | 0.0566 | 87.8 | < 0.05 |  |  |  |  |  |  |  |  |  |  |  |  |
| Phase Only | 5.09 | 0.0609 | 83.6 | < 0.05 |  |  |  |  |  |  |  |  | -0.496 | 0.121 | -4.09 | < 0.05 |
| Time Only | 5.08 | 0.0681 | 74.5 | < 0.05 | -0.00188 | 0.000712 | -2.64 | < 0.05 |  |  |  |  |  |  |  |  |
| Time + Phase | 5.06 | 0.0651 | 77.8 | < 0.05 | 0.00169 | 0.00127 | 1.33 | n.s. |  |  |  |  | -0.75 | 0.226 | -3.31 | < 0.05 |
| Time * Phase | 4.90 | 0.0784 | 62.5 | < 0.05 | 0.0104 | 0.00287 | 3.6 | < 0.05 | -0.0106 | 0.00317 | -3.33 | < 0.05 | -0.273 | 0.259 | -1.05 | n.s. |
| **1500 km summed MST distance** | | | | | | | | | | | | | | | | |
| Intercept Only | 5.09 | 0.0654 | 77.9 | < 0.05 |  |  |  |  |  |  |  |  |  |  |  |  |
| Phase Only | 5.19 | 0.0701 | 74.0 | < 0.05 |  |  |  |  |  |  |  |  | -0.482 | 0.155 | -3.1 | < 0.05 |
| Time Only | 5.17 | 0.0770 | 67.2 | < 0.05 | -0.00166 | 0.000894 | -1.85 | n.s. |  |  |  |  |  |  |  |  |
| Time + Phase | 5.16 | 0.0744 | 69.3 | < 0.05 | 0.00215 | 0.00161 | 1.33 | n.s. |  |  |  |  | -0.807 | 0.289 | -2.79 | < 0.05 |
| Time * Phase | 4.97 | 0.0888 | 55.9 | < 0.05 | 0.014 | 0.00373 | 3.75 | < 0.05 | -0.0142 | 0.00409 | -3.48 | < 0.05 | -0.223 | 0.32 | -0.696 | n.s. |
| **2000 km summed MST distance** | | | | | | | | | | | | | | | | |
| Intercept Only | 5.10 | 0.0655 | 77.8 | < 0.05 |  |  |  |  |  |  |  |  |  |  |  |  |
| Phase Only | 5.18 | 0.0743 | 69.8 | < 0.05 |  |  |  |  |  |  |  |  | -0.329 | 0.147 | -2.24 | < 0.05 |
| Time Only | 5.14 | 0.0813 | 63.2 | < 0.05 | -0.000777 | 0.000871 | -0.893 | n.s. |  |  |  |  |  |  |  |  |
| Time + Phase | 5.13 | 0.0786 | 65.3 | < 0.05 | 0.00273 | 0.00153 | 1.78 | n.s. |  |  |  |  | -0.724 | 0.265 | -2.73 | < 0.05 |
| Time * Phase | 4.97 | 0.0973 | 51.1 | < 0.05 | 0.0115 | 0.00364 | 3.17 | < 0.05 | -0.0106 | 0.00398 | -2.65 | < 0.05 | -0.276 | 0.307 | -0.9 | n.s. |
| **2500 km summed MST distance** | | | | | | | | | | | | | | | | |
| Intercept Only | 5.08 | 0.0723 | 70.2 | < 0.05 |  |  |  |  |  |  |  |  |  |  |  |  |
| Phase Only | 5.25 | 0.0797 | 65.8 | < 0.05 |  |  |  |  |  |  |  |  | -0.579 | 0.147 | -3.95 | < 0.05 |
| Time Only | 5.21 | 0.0863 | 60.3 | < 0.05 | -0.00218 | 0.000859 | -2.54 | < 0.05 |  |  |  |  |  |  |  |  |
| Time + Phase | 5.23 | 0.0827 | 63.2 | < 0.05 | 0.0013 | 0.0014 | 0.923 | n.s. |  |  |  |  | -0.767 | 0.251 | -3.05 | < 0.05 |
| Time * Phase | 4.98 | 0.0985 | 50.6 | < 0.05 | 0.017 | 0.00419 | 4.06 | < 0.05 | -0.0174 | 0.0044 | -3.94 | < 0.05 | -0.255 | 0.266 | -0.958 | n.s. |
| **3000 km summed MST distance** | | | | | | | | | | | | | | | | |
| Intercept Only | 5.20 | 0.0772 | 67.3 | < 0.05 |  |  |  |  |  |  |  |  |  |  |  |  |
| Phase Only | 5.35 | 0.0849 | 63.0 | < 0.05 |  |  |  |  |  |  |  |  | -0.548 | 0.164 | -3.35 | < 0.05 |
| Time Only | 5.33 | 0.0919 | 57.9 | < 0.05 | -0.00226 | 0.00094 | -2.41 | < 0.05 |  |  |  |  |  |  |  |  |
| Time + Phase | 5.33 | 0.0894 | 59.6 | < 0.05 | 0.00105 | 0.00169 | 0.618 | n.s. |  |  |  |  | -0.706 | 0.304 | -2.32 | < 0.05 |
| Time * Phase | 5.07 | 0.1040 | 48.5 | < 0.05 | 0.0174 | 0.00437 | 3.98 | < 0.05 | -0.0187 | 0.00467 | -4 | < 0.05 | -0.0546 | 0.322 | -0.17 | n.s. |
| **3500 km summed MST distance** | | | | | | | | | | | | | | | | |
| Intercept Only | 5.27 | 0.0773 | 68.3 | < 0.05 |  |  |  |  |  |  |  |  |  |  |  |  |
| Phase Only | 5.48 | 0.0831 | 66.0 | < 0.05 |  |  |  |  |  |  |  |  | -0.675 | 0.149 | -4.53 | < 0.05 |
| Time Only | 5.42 | 0.0926 | 58.6 | < 0.05 | -0.00235 | 0.000857 | -2.74 | < 0.05 |  |  |  |  |  |  |  |  |
| Time + Phase | 5.45 | 0.0856 | 63.7 | < 0.05 | 0.00205 | 0.00141 | 1.46 | n.s. |  |  |  |  | -0.993 | 0.264 | -3.76 | < 0.05 |
| Time * Phase | 5.21 | 0.1020 | 51.0 | < 0.05 | 0.0167 | 0.00416 | 4.02 | < 0.05 | -0.0163 | 0.00438 | -3.71 | < 0.05 | -0.479 | 0.28 | -1.71 | n.s. |
| **4000 km summed MST distance** | | | | | | | | | | | | | | | | |
| Intercept Only | 5.33 | 0.0859 | 62.1 | < 0.05 |  |  |  |  |  |  |  |  |  |  |  |  |
| Phase Only | 5.52 | 0.0960 | 57.5 | < 0.05 |  |  |  |  |  |  |  |  | -0.605 | 0.171 | -3.53 | < 0.05 |
| Time Only | 5.48 | 0.1050 | 52.4 | < 0.05 | -0.00214 | 0.000946 | -2.26 | < 0.05 |  |  |  |  |  |  |  |  |
| Time + Phase | 5.50 | 0.0999 | 55.0 | < 0.05 | 0.00163 | 0.00162 | 1.01 | n.s. |  |  |  |  | -0.861 | 0.307 | -2.8 | < 0.05 |
| Time * Phase | 5.23 | 0.1240 | 42.2 | < 0.05 | 0.0169 | 0.00499 | 3.39 | < 0.05 | -0.0168 | 0.00523 | -3.22 | < 0.05 | -0.328 | 0.332 | -0.988 | n.s. |

**Table S11.** Model selection using the second-order Akaike information criterion (AICc) to compare fits of linear models of spatially-standardised non-flying terrestrial species richness (Chao 2 extrapolated species richness) as a function of time and diversification phase.

| **model** | **df** | **logLik** | **AICc** | **delta AICc** | **weights** | **cumulative weights** | **evidence ratio** |
| --- | --- | --- | --- | --- | --- | --- | --- |
| **1000 km summed MST distance** | | | | | | | |
| Time * Phase | 4 | -71.9 | 154 | 0.00 | 8.40e-01 | 0.840 | 1.00e+00 |
| Phase Only | 2 | -76.2 | 159 | 4.44 | 9.14e-02 | 0.931 | 9.19e+00 |
| Time + Phase | 3 | -75.5 | 159 | 5.07 | 6.64e-02 | 0.998 | 1.27e+01 |
| Time Only | 2 | -80.1 | 166 | 12.30 | 1.82e-03 | 1.000 | 4.62e+02 |
| Intercept Only | 1 | -83.3 | 171 | 16.40 | 2.27e-04 | 1.000 | 3.70e+03 |
| **1500 km summed MST distance** | | | | | | | |
| Time * Phase | 4 | -72.1 | 155 | 0.00 | 9.91e-01 | 0.991 | 1.00e+00 |
| Time + Phase | 3 | -78.7 | 166 | 10.90 | 4.21e-03 | 0.995 | 2.35e+02 |
| Phase Only | 2 | -79.8 | 166 | 11.00 | 4.07e-03 | 0.999 | 2.43e+02 |
| Time Only | 2 | -82.4 | 171 | 16.10 | 3.10e-04 | 1.000 | 3.20e+03 |
| Intercept Only | 1 | -83.5 | 171 | 16.30 | 2.84e-04 | 1.000 | 3.49e+03 |
| **2000 km summed MST distance** | | | | | | | |
| Time * Phase | 4 | -71.2 | 153 | 0.00 | 9.43e-01 | 0.943 | 1.00e+00 |
| Time + Phase | 3 | -75.8 | 160 | 7.05 | 2.77e-02 | 0.971 | 3.40e+01 |
| Phase Only | 2 | -77.4 | 161 | 8.13 | 1.62e-02 | 0.987 | 5.82e+01 |
| Intercept Only | 1 | -79.0 | 162 | 9.23 | 9.36e-03 | 0.996 | 1.01e+02 |
| Time Only | 2 | -78.9 | 164 | 11.00 | 3.78e-03 | 1.000 | 2.49e+02 |
| **2500 km summed MST distance** | | | | | | | |
| Time * Phase | 4 | -67.5 | 146 | 0.00 | 9.99e-01 | 0.999 | 1.00e+00 |
| Time + Phase | 3 | -76.2 | 161 | 15.00 | 5.45e-04 | 1.000 | 1.83e+03 |
| Phase Only | 2 | -77.3 | 161 | 15.10 | 5.23e-04 | 1.000 | 1.91e+03 |
| Time Only | 2 | -81.3 | 169 | 23.30 | 8.90e-06 | 1.000 | 1.12e+05 |
| Intercept Only | 1 | -83.1 | 170 | 24.80 | 4.20e-06 | 1.000 | 2.39e+05 |
| **3000 km summed MST distance** | | | | | | | |
| Time * Phase | 4 | -64.4 | 139 | 0.00 | 9.88e-01 | 0.988 | 1.00e+00 |
| Phase Only | 2 | -71.6 | 149 | 9.97 | 6.76e-03 | 0.995 | 1.46e+02 |
| Time + Phase | 3 | -70.9 | 150 | 10.80 | 4.41e-03 | 0.999 | 2.24e+02 |
| Time Only | 2 | -74.6 | 155 | 16.00 | 3.29e-04 | 0.999 | 3.00e+03 |
| Intercept Only | 1 | -76.8 | 158 | 18.20 | 1.11e-04 | 1.000 | 8.90e+03 |
| **3500 km summed MST distance** | | | | | | | |
| Time * Phase | 4 | -53.3 | 117 | 0.00 | 9.94e-01 | 0.994 | 1.00e+00 |
| Time + Phase | 3 | -60.3 | 129 | 11.60 | 2.96e-03 | 0.997 | 3.36e+02 |
| Phase Only | 2 | -61.5 | 129 | 11.90 | 2.59e-03 | 1.000 | 3.84e+02 |
| Time Only | 2 | -67.3 | 141 | 23.50 | 7.70e-06 | 1.000 | 1.29e+05 |
| Intercept Only | 1 | -70.9 | 146 | 28.60 | 6.00e-07 | 1.000 | 1.62e+06 |
| **4000 km summed MST distance** | | | | | | | |
| Time * Phase | 4 | -57.8 | 126 | 0.00 | 9.43e-01 | 0.943 | 1.00e+00 |
| Phase Only | 2 | -63.4 | 133 | 6.79 | 3.17e-02 | 0.975 | 2.97e+01 |
| Time + Phase | 3 | -62.6 | 134 | 7.33 | 2.42e-02 | 0.999 | 3.90e+01 |
| Time Only | 2 | -67.0 | 140 | 14.00 | 8.71e-04 | 1.000 | 1.08e+03 |
| Intercept Only | 1 | -69.2 | 142 | 16.20 | 2.87e-04 | 1.000 | 3.29e+03 |

**Table S12.** Parameter estimates for coefficients in linear models fitted to spatially-standardised terrestrial tetrapod species richness data (Chao 2 extrapolated species richness). All models fitted to each palaeogeographic spread level are shown, regardless of Akaike weight, and ordering does not reflect importance.

|  | **Intercept** | | | | **Time** | | | | **Time : Phase (Pre-K/Pg)** | | | | **Phase (Pre-K/Pg)** | | | |
| --- | --- | --- | --- | --- | --- | --- | --- | --- | --- | --- | --- | --- | --- | --- | --- | --- |
| **model** | **estimate** | **std.error** | **statistic** | **p.value** | **estimate** | **std.error** | **statistic** | **p.value** | **estimate** | **std.error** | **statistic** | **p.value** | **estimate** | **std.error** | **statistic** | **p.value** |
| **1000 km summed MST distance** | | | | | | | | | | | | | | | | |
| Intercept Only | 5.07 | 0.0538 | 94.2 | < 0.05 |  |  |  |  |  |  |  |  |  |  |  |  |
| Phase Only | 5.18 | 0.0584 | 88.7 | < 0.05 |  |  |  |  |  |  |  |  | -0.447 | 0.116 | -3.85 | < 0.05 |
| Time Only | 5.16 | 0.0649 | 79.5 | < 0.05 | -0.00171 | 0.000679 | -2.51 | < 0.05 |  |  |  |  |  |  |  |  |
| Time + Phase | 5.15 | 0.0624 | 82.5 | < 0.05 | 0.00147 | 0.00122 | 1.2 | n.s. |  |  |  |  | -0.668 | 0.217 | -3.08 | < 0.05 |
| Time * Phase | 5.03 | 0.0766 | 65.6 | < 0.05 | 0.00831 | 0.00281 | 2.96 | < 0.05 | -0.00832 | 0.0031 | -2.68 | < 0.05 | -0.292 | 0.253 | -1.15 | n.s. |
| **1500 km summed MST distance** | | | | | | | | | | | | | | | | |
| Intercept Only | 5.19 | 0.0619 | 83.8 | < 0.05 |  |  |  |  |  |  |  |  |  |  |  |  |
| Phase Only | 5.27 | 0.0671 | 78.6 | < 0.05 |  |  |  |  |  |  |  |  | -0.408 | 0.148 | -2.75 | < 0.05 |
| Time Only | 5.25 | 0.0733 | 71.6 | < 0.05 | -0.00128 | 0.000852 | -1.5 | n.s. |  |  |  |  |  |  |  |  |
| Time + Phase | 5.23 | 0.0710 | 73.7 | < 0.05 | 0.00226 | 0.00154 | 1.47 | n.s. |  |  |  |  | -0.75 | 0.276 | -2.72 | < 0.05 |
| Time * Phase | 5.05 | 0.0842 | 59.9 | < 0.05 | 0.0141 | 0.00354 | 3.99 | < 0.05 | -0.0142 | 0.00388 | -3.67 | < 0.05 | -0.166 | 0.303 | -0.546 | n.s. |
| **2000 km summed MST distance** | | | | | | | | | | | | | | | | |
| Intercept Only | 5.16 | 0.0617 | 83.6 | < 0.05 |  |  |  |  |  |  |  |  |  |  |  |  |
| Phase Only | 5.23 | 0.0707 | 73.9 | < 0.05 |  |  |  |  |  |  |  |  | -0.249 | 0.14 | -1.78 | n.s. |
| Time Only | 5.19 | 0.0768 | 67.5 | < 0.05 | -0.000429 | 0.000822 | -0.521 | n.s. |  |  |  |  |  |  |  |  |
| Time + Phase | 5.18 | 0.0748 | 69.3 | < 0.05 | 0.0026 | 0.00146 | 1.78 | n.s. |  |  |  |  | -0.625 | 0.252 | -2.48 | < 0.05 |
| Time * Phase | 5.00 | 0.0914 | 54.7 | < 0.05 | 0.0121 | 0.00342 | 3.54 | < 0.05 | -0.0114 | 0.00374 | -3.05 | < 0.05 | -0.141 | 0.289 | -0.487 | n.s. |
| **2500 km summed MST distance** | | | | | | | | | | | | | | | | |
| Intercept Only | 5.16 | 0.0667 | 77.3 | < 0.05 |  |  |  |  |  |  |  |  |  |  |  |  |
| Phase Only | 5.30 | 0.0748 | 70.8 | < 0.05 |  |  |  |  |  |  |  |  | -0.482 | 0.138 | -3.51 | < 0.05 |
| Time Only | 5.25 | 0.0809 | 64.8 | < 0.05 | -0.00153 | 0.000805 | -1.9 | n.s. |  |  |  |  |  |  |  |  |
| Time + Phase | 5.27 | 0.0770 | 68.4 | < 0.05 | 0.00193 | 0.00131 | 1.48 | n.s. |  |  |  |  | -0.762 | 0.234 | -3.26 | < 0.05 |
| Time * Phase | 5.03 | 0.0905 | 55.5 | < 0.05 | 0.0175 | 0.00385 | 4.55 | < 0.05 | -0.0172 | 0.00405 | -4.26 | < 0.05 | -0.253 | 0.244 | -1.04 | n.s. |
| **3000 km summed MST distance** | | | | | | | | | | | | | | | | |
| Intercept Only | 5.26 | 0.0738 | 71.3 | < 0.05 |  |  |  |  |  |  |  |  |  |  |  |  |
| Phase Only | 5.40 | 0.0813 | 66.4 | < 0.05 |  |  |  |  |  |  |  |  | -0.514 | 0.157 | -3.28 | < 0.05 |
| Time Only | 5.36 | 0.0886 | 60.5 | < 0.05 | -0.00188 | 0.000907 | -2.07 | < 0.05 |  |  |  |  |  |  |  |  |
| Time + Phase | 5.37 | 0.0851 | 63.1 | < 0.05 | 0.00182 | 0.00161 | 1.13 | n.s. |  |  |  |  | -0.789 | 0.29 | -2.72 | < 0.05 |
| Time * Phase | 5.14 | 0.1010 | 50.9 | < 0.05 | 0.0163 | 0.00422 | 3.86 | < 0.05 | -0.0166 | 0.00451 | -3.67 | < 0.05 | -0.211 | 0.311 | -0.68 | n.s. |
| **3500 km summed MST distance** | | | | | | | | | | | | | | | | |
| Intercept Only | 5.37 | 0.0697 | 77.1 | < 0.05 |  |  |  |  |  |  |  |  |  |  |  |  |
| Phase Only | 5.56 | 0.0748 | 74.3 | < 0.05 |  |  |  |  |  |  |  |  | -0.611 | 0.134 | -4.55 | < 0.05 |
| Time Only | 5.51 | 0.0836 | 65.9 | < 0.05 | -0.00209 | 0.000773 | -2.71 | < 0.05 |  |  |  |  |  |  |  |  |
| Time + Phase | 5.53 | 0.0770 | 71.8 | < 0.05 | 0.00196 | 0.00127 | 1.54 | n.s. |  |  |  |  | -0.914 | 0.237 | -3.85 | < 0.05 |
| Time * Phase | 5.31 | 0.0916 | 58.0 | < 0.05 | 0.0154 | 0.00373 | 4.13 | < 0.05 | -0.0149 | 0.00393 | -3.79 | < 0.05 | -0.443 | 0.251 | -1.76 | n.s. |
| **4000 km summed MST distance** | | | | | | | | | | | | | | | | |
| Intercept Only | 5.43 | 0.0783 | 69.4 | < 0.05 |  |  |  |  |  |  |  |  |  |  |  |  |
| Phase Only | 5.60 | 0.0877 | 63.9 | < 0.05 |  |  |  |  |  |  |  |  | -0.546 | 0.156 | -3.49 | < 0.05 |
| Time Only | 5.55 | 0.0959 | 57.9 | < 0.05 | -0.00181 | 0.000867 | -2.09 | < 0.05 |  |  |  |  |  |  |  |  |
| Time + Phase | 5.57 | 0.0909 | 61.3 | < 0.05 | 0.00186 | 0.00147 | 1.26 | n.s. |  |  |  |  | -0.839 | 0.279 | -3 | < 0.05 |
| Time * Phase | 5.34 | 0.1130 | 47.2 | < 0.05 | 0.0154 | 0.00455 | 3.37 | < 0.05 | -0.0149 | 0.00478 | -3.11 | < 0.05 | -0.368 | 0.303 | -1.21 | n.s. |
